# Hitting an Unintended Target: Phylogeography of *Bombus brasiliensis* Lepeletier, 1836 and the First New Brazilian Bumblebee Species in a Century (Hymenoptera: Apidae)

**DOI:** 10.1371/journal.pone.0125847

**Published:** 2015-05-20

**Authors:** José Eustáquio Santos Júnior, Fabrício R. Santos, Fernando A. Silveira

**Affiliations:** 1 Departamento de Biologia Geral, Instituto de Ciências Biológicas, Universidade Federal de Minas Gerais, Belo Horizonte, Minas Gerais, Brazil; 2 Departamento de Zoologia, Instituto de Ciências Biológicas, Universidade Federal de Minas Gerais, Belo Horizonte, Minas Gerais, Brazil; Smithsonian National Museum of Natural History, UNITED STATES

## Abstract

This work tested whether or not populations of *Bombus brasiliensis* isolated on mountain tops of southeastern Brazil belonged to the same species as populations widespread in lowland areas in the Atlantic coast and westward along the Paraná-river valley. Phylogeographic and population genetic analyses showed that those populations were all conspecific. However, they revealed a previously unrecognized, apparently rare, and potentially endangered species in one of the most threatened biodiversity hotspots of the World, the Brazilian Atlantic Forest. This species is described here as *Bombus bahiensis* sp. n., and included in a revised key for the identification of the bumblebee species known to occur in Brazil. Phylogenetic analyses based on two *mt*DNA markers suggest this new species to be sister to *B*. *brasiliensis*, from which its workers and queens can be easily distinguished by the lack of a yellow hair-band on the first metasomal tergum. The results presented here are consistent with the hypothesis that *B*. *bahiensis* sp. n. may have originated from an ancestral population isolated in an evergreen-forest refuge (the so-called Bahia refuge) during cold, dry periods of the Pleistocene. This refuge is also known as an important area of endemism for several animal taxa, including other bees. Secondary contact between *B*. *bahiensis* and *B*. *brasiliensis* may be presently prevented by a strip of semi-deciduous forest in a climate zone characterized by relatively long dry seasons. Considering the relatively limited range of this new species and the current anthropic pressure on its environment, attention should be given to its conservation status.

## Introduction

Despite the fact that, taxonomically, bees are among the best known insects in the world (for example [1]), there are taxonomic problems to be solved in the group, including sex association in dimorphic species and distinction of cryptic species, even in relatively well-known taxa. In these cases, molecular data, including the so called DNA barcode, alone or integrated to other types of characters, have proven to be much useful (for example [2, 3]).

The bumblebees (genus *Bombus* Latreille, 1802) comprise approximately 250 species widely distributed in the world, but occurring mainly in the cool subtropical and temperate areas of the Nearctic and, especially, Palearctic regions [4]. Defining species limits in *Bombus* is frequently difficult, because of the lack of useful structural characters and the great variability in hair-color patterns. This has led to an array of recent studies employing DNA markers to elucidate bumblebee-species boundaries (for example [5–12]). Molecular tools have also been used in the genus to investigate intraspecific genetic structure, phylogeography and phylogenetic relationships (for example [13–22]).

In South America, most of the species of *Bombus* are distributed along the Andes and in temperate regions, with only a few species recorded in the warm lowlands—the later, actually, the only bumblebees to occur in such environments in the world [1]. Only six species of the genus are generally referred to occur in Brazil [23], all belonging to the same subgenus—*Fervidobombus* Skorikov, 1922 or *Thoracobombus* Dalla Torre, 1880, depending on which classification one adopts (for example [1, 24] or [25]). Five of these species (*B*. *bellicosus* Smith, 1879; *B*. *brasiliensis* Lepeletier, 1836; *B*. *brevivillus* Franklin, 1913; *B*. *pauloensis* Friese, 1913; and *B*. *transversalis* (Olivier, 1789)) seem to be very closely related, while the sixth one, *B*. *morio* (Swederus, 1787) belongs to a distinctive clade in the same subgenus [19, 21]. *Bombus pauloensis* has been widely treated as *B*. *atratus* Franklin, 1913 in the literature (for an explanation for the adoption of *B*. *pauloensis* as a valid name, see [24]). The occurrence of a seventh species in western Brazil, *B*. *pullatus* Franklin, 1913, was reported by Milliron [26], but considered with suspicion by Abrahamovich & Díaz [27], and ignored by Moure & Melo [24], and needs confirmation. An eighth species, *B*. *rubriventris* Lepeletier, 1836, was recorded with doubts by Milliron [26] as possibly occurring in the state of Goiás. According to him, this species, known only from its female holotype, would be very rare or extinct. One cannot rule out the possibility that the assignment of *B*. *rubriventris* to Brazil resulted from mere labeling mistake or from wrong interpretation of the type locality (see [26] for details).

In their thorough revision of the Brazilian bumblebees, Moure & Sakagami [23]) noted that *B*. *brasiliensis* Lepeletier, 1836 was the only species to be more common on mountaintops (mainly in the ranges along the southern and eastern Brazilian coasts) than in lowlands, and that it was the only one recorded in elevations above 1,800 m. Later, Silveira & Cure [28] noted that *B*. *brasiliensis* is absent in the lowlands surrounding the mountain ranges where it is relatively common further inland in southeastern Brazil. The occurrence of these isolated mountaintop populations of *B*. *brasiliensis* raised the suspicion that they might in fact belong to a species different from the one present in the lowlands along the coast and in the Paraná River valley. Here, this hypothesis is tested, mainly by means of phylogenetic and population-genetic analyses of two *mt*DNA genes. Since the question of how to delimit species boundaries is still in debate (see, for instance, the revision by Wiley & Lieberman [29]), different character sources (molecular and morphological) and analytical methods (tree and non-tree based) were complementarily employed to decide whether or not a set of populations should be considered as a new taxon.

## Materials and Methods

### Morphological and Molecular Procedures

The description of the new species presented below was based on six specimens—three workers and a queen from the municipality of Ilhéus, in the Brazilian state of Bahia, and two workers collected in the municipality of Conceição da Barra, state of Espírito Santo. These specimens are deposited at the Taxonomic Collections of the ‘Universidade Federal de Minas Gerais’—UFMG—and in the ‘Padre Jesus Santiago Moure’ entomological collection, of the ‘Universidade Federal do Paraná’—DZUP—, as detailed in the section “Taxonomic treatment”, below. Two collecting expeditions were conducted in southern Bahia and northern Espírito Santo states (coordinates of sampling sites are, in Bahia: Eunápolis—16°25'8"S, 39°34'55"W, 162m; Itamaraju—16°58'39"S, 39°33'16"W, 81m; Porto Seguro—16°27'3"S, 39°17'16"W, 114m; and, in Espírito Santo: Conceição da Barra—18°20'54"S,39°51'06"W, 47m; São Mateus—18°45'13"S, 39°51'39"W, 39 m; Sooretama—19°03'01"S, 40°08'02"W, 94m), under the collecting permit number 23784 (granted by “Instituto Chico Mendes de Conservação da Biodiversidade” to JESJ), in an attempt to increase molecular sample size and geographic representation. No additional specimens were found, however, during these expeditions.

**Fig 1:**
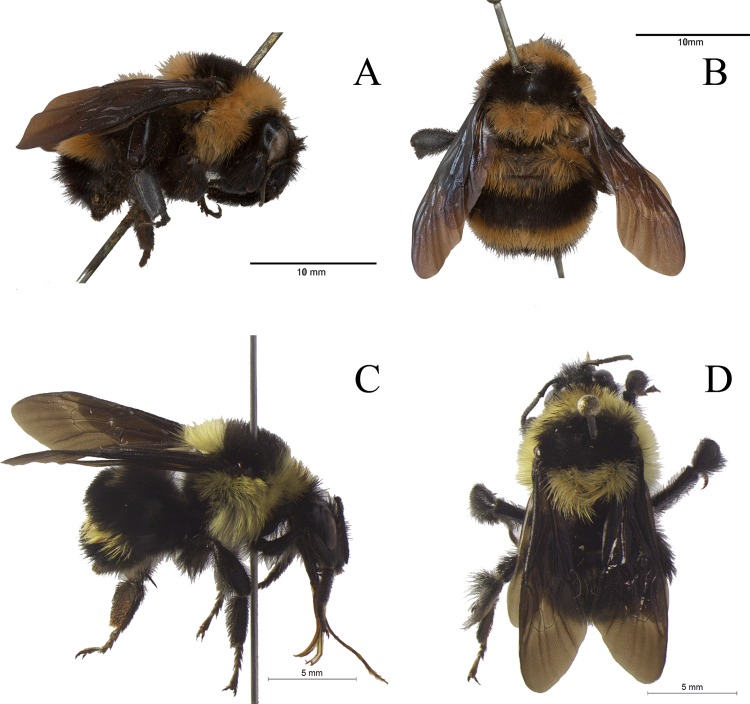
Types of *Bombus brasiliensis* Lepeletier, 1836 and *Bombus bahiensis* sp. n. Lectotype of *B*. *brasiliensis*—A: lateral view of head, mesosoma and metasoma; B: dorsal view of mesosoma and metasoma. Holotype of *B*. *bahiensis* sp. n.—C: lateral view of head, mesosoma and metasoma; D: dorsal view of mesosoma and metasoma.

The specimens were examined under a dissection microscope (Leica M125) and compared to the descriptions in Moure & Sakagami [23]. Comparisons were also made with photographs of the lectotype of *B*. *brasiliensis* Lepeletier, 1836, reproduced here in Fig 1A and 1B. Direct observation of this type was not done because, after examining hundreds of specimens of *B*. *brasiliensis* from all its geographic range, an easily-observable diagnostic character was found to distinguish the two concerned species, which could be readily checked in the photographs obtained from the type. Morphological terminology employed here is mostly that of Moure & Sakagami [23]. Flagellomeres are designated as F1, F2, F3 etc. Accordingly, metasomal terga and sterna are designated as T1, T2, T3 etc and S1, S2, S3 etc. The apical width of the malar area is the shortest width of the malar area, measured along the mandible base.

Two mitochondrial markers, Cytochrome oxidase I (COI) and Cytochrome B (CytB) were employed in addition to the morphological characters. DNA was extracted from one of the hind legs of each of 168 *Bombus* specimens, 142 of them belonging to *B*. *brasiliensis*, three to the species described as new below, and the rest belonging to the other species of the genus known to occur in Brazil (S1 Table). This was done through the phenol–chloroform method [30]. The extracted DNA was re-suspended in 40μL of TE buffer. The 5’region of the COI gene was amplified using the primers LepF1 and LepR1 [31], mtd_6 and mtd_9 [32], and that of the CytB was amplified using the primers mtd_26 [32] and CytB_R1, 5'TTCAATTATTTGACTTCCTAATCAAG3' (designed for this analysis). Amplification of both genes was done in a 30μL polymerase chain reaction (PCR) mix, including 0.5 units of Taq DNA polymerase, 2 mM MgCl_2_, in 1x PCR buffer, 0.5 μM of each primer, 2.5 mMdNTPs and about 20 ng of genomic DNA. Amplification was carried out in a thermocycler using a program consisting of 5 min of denaturation at 94°C, followed by 35 30-second cycles at 94°C, 40 s at 48–51°C, 1 min at 72°C, and a final extension for 10 min at 72°C. PCR products were visualized in a 0.8% agarose gel. All PCRs that generated a single product were purified using polyethylene glycol 20% (PEG) precipitation (modified of Sambrook & Russel [30]—S2 Table). This purified PCR product was sequenced in both directions using a MegaBACE 1000 sequencer (GE Healthcare) with the same amplification primers following the manufacturer's recommendations. The raw sequences were obtained using the software Phred v. 0.20425 [33]. The final sequences were assembled with the software Phrap v. 0.990319 (http//www.phrap.org). Consed 19.0 [34] was used to view and edit the high quality consensus sequences. Alignments of the consensus sequences for all individuals were generated using MUSCLE [35] implemented in the program MEGA 5.01 [36].

Molecular extraction and sequencing were done in the Biodiversity and Molecular Evolution Lab (LBEM), at the Universidade Federal de Minas Gerais, Belo Horizonte, Brazil. In addition to the 306 sequences generated here (168 COI, and 138 CytB sequences) for five species, 49 COI sequences available in the GenBank were employed for 16 species belonging to the subgenus *Thoracobombus* (sensu Williams et al. [25]) (Table 1). Additionally, sequences from three others specimens of *B*. *bellicosus*, stored in the Barcode of Life Database, and kindly made available by Dr. P. Tubaro, from the Museo Argentino de Ciencias Naturales, were also employed (Table 1).

**Table 1:** Specimens belonging to GenBank and BOLD Systems databases, which were used in analyzes with the COI gene.

Accession number	Species	Database
KC853321.1	*Bombus excellens* Smith, 1879	GenBank
KC853363.1	*Bombus medius* Cresson, 1863	GenBank
KC853356.1	*Bombus pauloensis* Friese, 1913	GenBank
KC853357.1	*Bombus pauloensis* Friese, 1913	GenBank
KC853358.1	*Bombus pauloensis* Friese, 1913	GenBank
KC853359.1	*Bombus pauloensis* Friese, 1913	GenBank
KC853360.1	*Bombus pauloensis* Friese, 1913	GenBank
KC853361.1	*Bombus pensylvanicus* (De Geer, 1773)	GenBank
ARG-06832-75	*Bombus bellicosus* Smith, 1879	BOLDSYSTEMS
ARG-06832-76	*Bombus bellicosus* Smith, 1879	BOLDSYSTEMS
ARG-7205-37	*Bombus bellicosus* Smith, 1879	BOLDSYSTEMS
AF385820.1	*Bombus deuteronymus* Schulz, 1906	GenBank
KC853366_1	*Bombus diligens* Smith, 1861	GenBank
FJ582118.1	*Bombus fervidus* (Fabricius, 1798)	GenBank
FJ582119.1	*Bombus fervidus* (Fabricius, 1798)	GenBank
FJ582120.1	*Bombus fervidus* (Fabricius, 1798)	GenBank
FJ582122.1	*Bombus fervidus* (Fabricius, 1798)	GenBank
AY181106.1	*Bombus humilis* Illiger, 1806	GenBank
AY181127.1	*Bombus mesomelas* Gerstäcker, 1869	GenBank
AY181128.1	*Bombus mesomelas* Gerstäcker, 1869	GenBank
DQ225325.1	*Bombus morio* (Swederus, 1787)	GenBank
KC853367.1	*Bombus morio* (Swederus, 1787)	GenBank
KC853368.1	*Bombus morio* (Swederus, 1787)	GenBank
KC853369.1	*Bombus morio* (Swederus, 1787)	GenBank
KC853370.1	*Bombus morio* (Swederus, 1787)	GenBank
KC853371.1	*Bombus morio* (Swederus, 1787)	GenBank
AY181133.1	*Bombus muscorum* (Linnaeus, 1758)	GenBank
AY181134.1	*Bombus muscorum* (Linnaeus, 1758)	GenBank
AY181135.1	*Bombus muscorum* (Linnaeus, 1758)	GenBank
KC853365.1	*Bombus opifex* Smith, 1879	GenBank
AY181136.1	*Bombus pascuorum* (Scopoli, 1763)	GenBank
AY181137.1	*Bombus pascuorum* (Scopoli, 1763)	GenBank
AY181138.1	*Bombus pascuorum* (Scopoli, 1763)	GenBank
AY181139.1	*Bombus pascuorum* (Scopoli, 1763)	GenBank
AY181140.1	*Bombus pascuorum* (Scopoli, 1763)	GenBank
AY181141.1	*Bombus pascuorum* (Scopoli, 1763)	GenBank
AY181142.1	*Bombus pascuorum* (Scopoli, 1763)	GenBank
AY181143.1	*Bombus pascuorum* (Scopoli, 1763)	GenBank
JQ909709.1	*Bombus pascuorum* (Scopoli, 1763)	GenBank
JQ909710.1	*Bombus pascuorum* (Scopoli, 1763)	GenBank
AY181152.1	*Bombus ruderarius* (Müller, 1776)	GenBank
AY181153.1	*Bombus ruderarius* (Müller, 1776)	GenBank
AY181154.1	*Bombus ruderarius* (Müller, 1776)	GenBank
AY181155.1	*Bombus ruderarius* (Müller, 1776)	GenBank
AF385821.1	*Bombus schrencki* Morawitz, 1881	GenBank
GU674500.1	*Bombus schrencki* Morawitz, 1881	GenBank
AY181166.1	*Bombus sylvarum* Linnaeus, 1761	GenBank
AY181167.1	*Bombus sylvarum* Linnaeus, 1761	GenBank
AY181168.1	*Bombus sylvarum* Linnaeus, 1761	GenBank

The computer programs Arlequin version 3.5.1.2 [37], DnaSP version 5.10.01 [38] and MEGA 5.01 were used to estimate the following intra and interpopulational parameters: 1) haplotype diversity (H); 2) average number of nucleotide differences (k); 3) mean number of pairwise differences (π); 4) number of polymorphic sites (S); and 5) Tajima’s D and Fu’s F_S_ test of selective neutrality. DnaSP was also used to verify non-synonymous and synonymous substitutions. Haplotype networks constructed using the median-joining algorithm (MJ) [39], available in the NETWORK 4.5 software, were used for inferences about phylogenetic relationships among haplotypes and their possible geographical correlation. Population analyses were performed separately for *B*. *brasiliensis* (124 specimens) and *B*. *bahiensis* sp. n. (3 specimens) using a CytB and COI concatenated matrix (127 specimens).

Average intra and interspecific genetic distances were obtained with Mega 5.01, using the parameters of the Kimura 2 model—K2P [40]. Two analyses were performed with different data sets, one using only the COI gene and the other employing a concatenated data set of the COI and CytB genes. This was done because: 1) There were more COI than CytB sequences available in GenBank and BOLD for species of *Thoracobombus* (sensu Williams et al.[25]); and 2) There were no CytB sequences available for many of the specimens from which DNA was extracted for this work.

Since primers used here were not the same as those employed for obtaining the sequences in GenBank and BOLD, only 402 bp were present in all sequences used in the COI-only analyses. Intraspecific distances were estimated only for species represented by three or more specimens, with at least two different haplotypes.

Phylogenetic analyses were performed using a concatenated matrix with CytB and COI-gene data, including a total of 138 specimens, with *B*. *morio* as the outgroup. The best fit substitution model estimated with Modeltest 3.7 [41] for this analysis was the GTR+G model.

Phylogenies were generated through Bayesian Inference (BI) in MrBayes 3.1 [42], and through Maximum Parsimony (MP) and Maximum Likelihood (ML) algorithms using the program PAUP* 4.0b10 [43]. Inferences of trees with MP and ML methods were performed using heuristic search, with the following parameters: stepwise addition (random) starting from a single initial tree and branch swapping (tree bisection and reconnection—TBR). MP analysis was set to retain up to 1000 most parsimonious trees. The bootstrap method was used as a measure of branch support for the recovered phylogenies, using a total of 10,000 and 100 replications, respectively, for MP and ML. Phylogenetic analyses using BI used two sets of Markov chains, each containing three hot chains and one cold, with 20 million generations with a 25% burn-in, to seek for convergence to the same subset of best trees.

### Nomenclatural Acts

The electronic edition of this article conforms to the requirements of the amended International Code of Zoological Nomenclature, and hence the new names contained herein are available under that Code from the electronic edition of this article. This published work and the nomenclatural acts it contains have been registered in ZooBank, the online registration system for the ICZN. The ZooBank LSIDs (Life Science Identifiers) can be resolved and the associated information viewed through any standard web browser by appending the LSID to the prefix "http://zoobank.org/". The LSID for this publication is: urn:lsid:zoobank.org:act:D4CB4F65-BA90-4E9B-BFCF-F420A9718C25. The electronic edition of this work was published in a journal with an ISSN, and has been archived and is available from the following digital repositories: PubMed Central, LOCKSS.

## Results

### Concatenated population analyses

**Fig 2:**
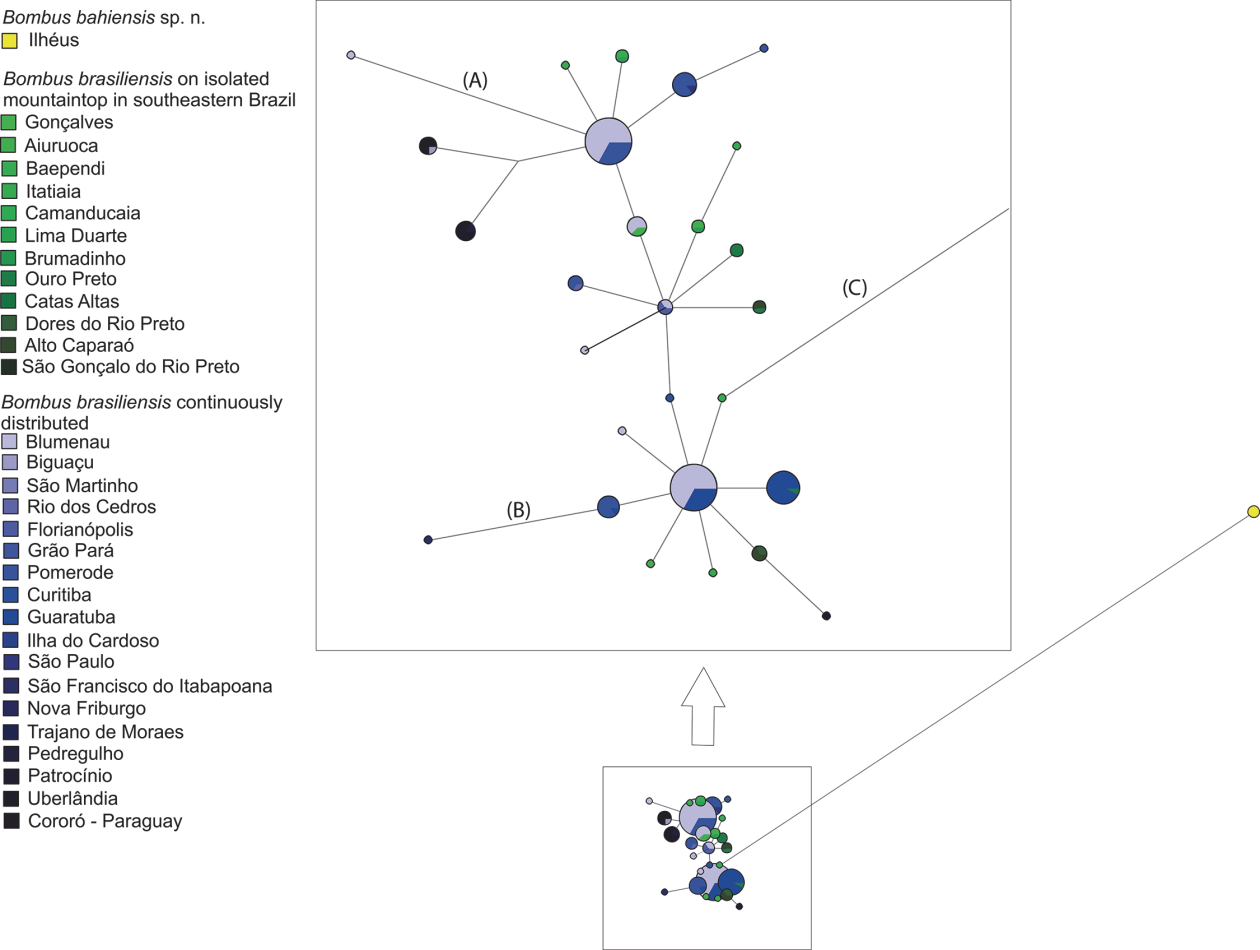
Network of haplotypes for *Bombus brasiliensis* Lepeletier, 1836 and *Bombus bahiensis* sp. n. Median-joining network of haplotypes for *B*. *brasiliensis* and *B*.*bahiensis* sp. n. The *B*. *brasiliensis* on isolated moutaintops in southeastern Brazil are coloured of different shades of green and the *B*. *brasiliensis* continuously distributed are coloured of different shades of blue. *B*.*bahiensis* sp. n. is coloured yellow. All lines joining haplotypes are one mutation step long, except for three of them, marked with “a”, “b” and “c”, which are three, two and 38 steps long, respectively.

The phylogenetic relationships among haplotypes and their geographical correlation, using the concatenated CytB (683 bp) and COI (471 bp) sequences (totaling 1154 bp), showed that populations from almost all the geographic range of *B*. *brasiliensis* share haplotypes, as shown by the haplotype network, which suggests lack of population structure (Fig 2). In contradiction to the initial hypothesis, thus, this result suggests that populations of *B*. *brasiliensis* isolated in mountain tops away from the coast belong in the same species as those living in coastal forests from the state of Rio de Janeiro southward to the state of Santa Catarina and westward to Paraguay in the Paraná-river valley.

On the other hand, these results indicate that the three specimens collected in the coastal forest of southern Bahia state (municipality of Ilhéus, all with the same haplotype) diverged from *B*. *brasiliensis* by 38 mutation steps (Fig 2), suggesting that this population belongs to a different species (described below as *B*. *bahiensis* sp. n.)

**Fig 3:**
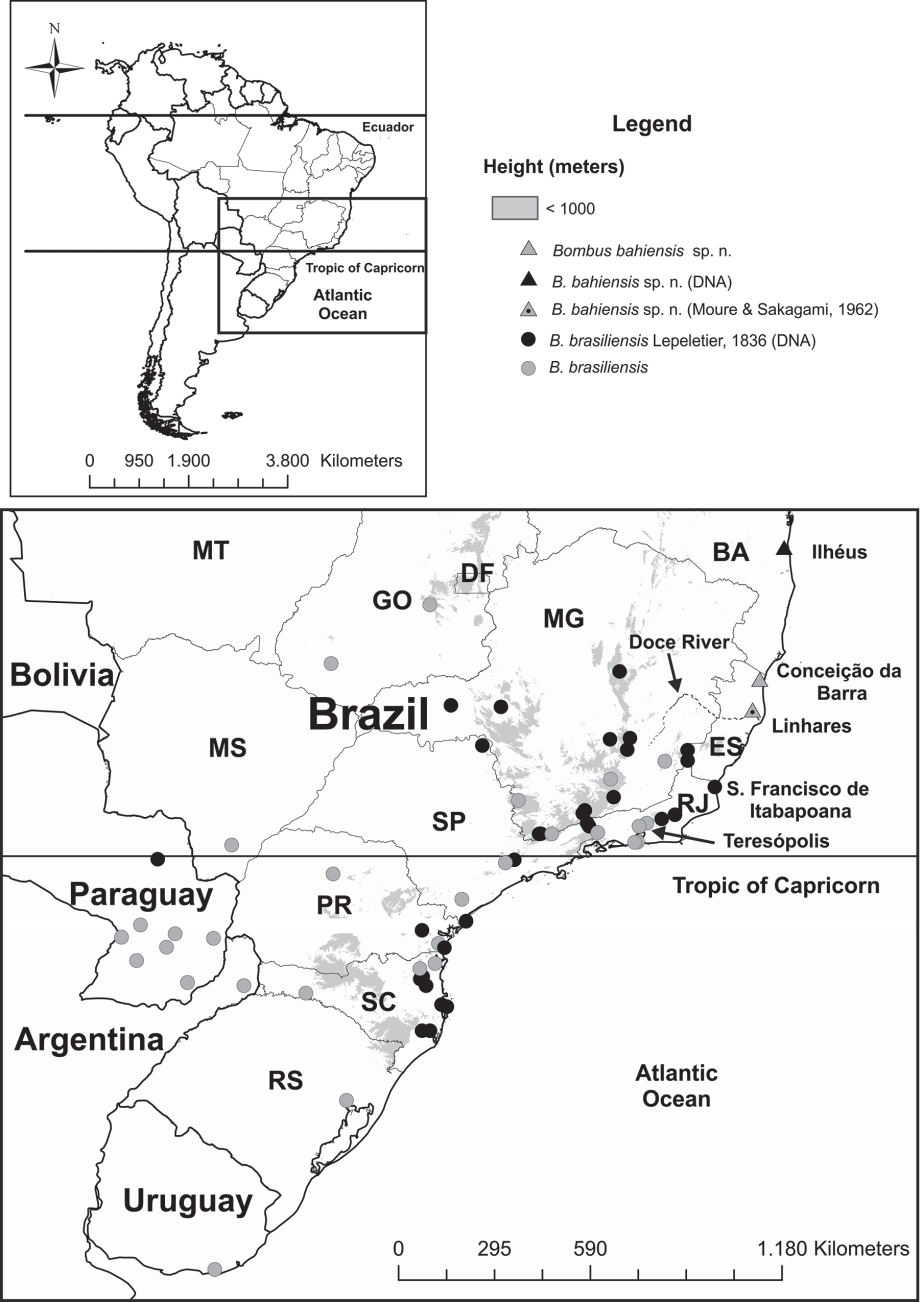
Distribution of *Bombus brasiliensis* Lepeletier, 1836 and *Bombus bahiensis* sp. n. Records for *B*. *bahiensis* are represented by triangles (the black triangle, the gray triangle and the gray triangle with black point represents a population used in mtDNA analyzed, a population used in morphologic analyzed and a population inferred to belong to the species based on descriptions in Moure &Sakagami [23], respectively); records for *B*. *brasiliensis* are represented by circles (the black circle denote sites represented by mtDNA). Records were compiled from the literature [23, 60, 73] and from specimens deposited at UFMG and/or listed in S1 Table. Acronyms represent Brazilian states, as follows: BA = Bahia; ES = Espírito Santo; MG = Minas Gerais; RJ = Rio de Janeiro; SP = São Paulo; PR = Paraná; SC = Santa Catarina; RS = Rio Grande do Sul; MT = Mato Grosso; GO = Goiás; MS = Mato Grosso do Sul; DF = Distrito Federal. Localities mentioned in the text are indicated. The stippled line indicated by black arrow represents the Doce river.

The population analysis of *B*. *brasiliensis* included a total of 124 specimens, from 30 localities (Fig 3). It resulted in 27 haplotypes, 17 polymorphic sites (11 synonymous and six nonsynonymous mutations), 10 of which were parsimoniously informative and seven were autapomorphies (singleton sites). The total haplotype diversity (Hd), nucleotide diversity (π), and average number of nucleotide differences (K) were 0.886, 0.003 and 3.057, respectively. The statistics for the neutrality tests were D = -0.083 (p = 0.5) for Tajima’s, and FS = -12.28 (p = 0.001) for Fu’s. Although the Tajima’s D was not statistically significant, the significant value of Fu’s FS, associated with the large number of unique haplotypes and the star-shaped haplotype network, suggests recent population expansion.

The genetic distance between *B*. *bahiensis* sp. n. and *B*. *brasiliensis* was 3.7 ± 0.6%, while the average intraspecific distance within *B*. *brasiliensis* was 0.26 ± 0.09%. The population parameters above were not calculated for *B*. *bahiensis* sp. n., since all three specimens had the same haplotype.

### COI-only analyses

Of the 402 bp used in the COI-only analyses, 241 were constant and, among the variable characters, 28 were parsimony-uninformative, and 133 parsimony-informative. The mean genetic distance between *B*. *bahiensis* sp. n. and *B*. *brasiliensis* estimated with the COI gene was 2.89 ± 0.8%. Among the comparisons made for the 21 *Thoracobombus* species employed in this analysis, distances smaller than that between *B*. *brasiliensis* and *B*. *bahiensis* were obtained for seven other species pairs (S3 Table). Moreover, while the largest distance between any two specimens of *B*. *brasiliensis* was of 1 ± 0.5%, the smallest distance between a specimen of *B*. *brasiliensis* and a specimen of *B*. *bahiensis* was of 2.5± 0.8%.

The intraspecific distances among the *Thoracobombus* species analyzed here, estimated with the COI sequences, were all equal or smaller than 0.5% (Table 2). One exception was the mean intraspecific distance for *B*. *brevivillus*, which was very high when all specimens originally attributed to this species where considered together (Table 2). Moreover, the average distance between the two “*B*. *brevivillus”* clades obtained in the phylogenetic results described below is 3.92 ± 0.93%. This suggests that the samples considered here as belonging to *B*. *brevivillus* include specimens of two distinct species (*B*. *brevivillus* (1) and (2) in S3 Table). This finding is being examined in detail and results will be published elsewhere.

**Table 2:** Genetic distances for COI sequences (%) within species of bumblebees.

Species	Average distance	MaD
*B*. *pauloensis*	0.46±0.17	1.51±0.60
*B*. *brasiliensis*	0.39±0.21	1.01±0.50
*B*. *morio*	0.35±0.14	1.77±0.67
*B*. *bahiensis* sp. n.	0.00±0.00	0.00±0.00
*B*. *transversalis*	0.10±0.10	0.25±0.24
*B*. *bellicosus*	0.50±0.29	0.75±0.43
*B*. *brevivillus* (2)	0.50±0.28	0.75±0.43
*B*. gr. *Brevivillus*	2.50±0.60	4.09±0.99
*B*. *muscorum*	0.00±0.00	0.00±0.00
*B*. *fervidus*	0.00±0.00	0.00±0.00
*B*. *pascuorum*	0.14±0.10	0.50±0.34
*B*. *ruderarius*	0.00±0.00	0.00±0.00

Analysis was done in species with three or more specimens. The model used was the Kimura 2-parameter. *B*. *brevivillus* was considered as belonging in two distinct species (*B*. *brevivillus* cluster 1 and cluster 2 see S3 Table) and *B*. gr. *brevivillus* (all specimens). AD = average intraspecific distance ± standard deviation; MaD = maximum intraspecific distance ± standard deviation.

### Phylogenetic analyses

Of the 1157 bp employed in the phylogenetic analyses (686 bp for CytB and 471 for COI), 916 were constant, three were gaps; eight variable characters were parsimony-uninformative, and 233 were parsimony-informative. Those three gaps are due to one CytB codon appearing exclusively in *B*. *morio* (included as the outgroup) and were the cause for the difference in pair-base numbers between the data employed in the phylogenetic and the population analyses.

**Fig 4:**
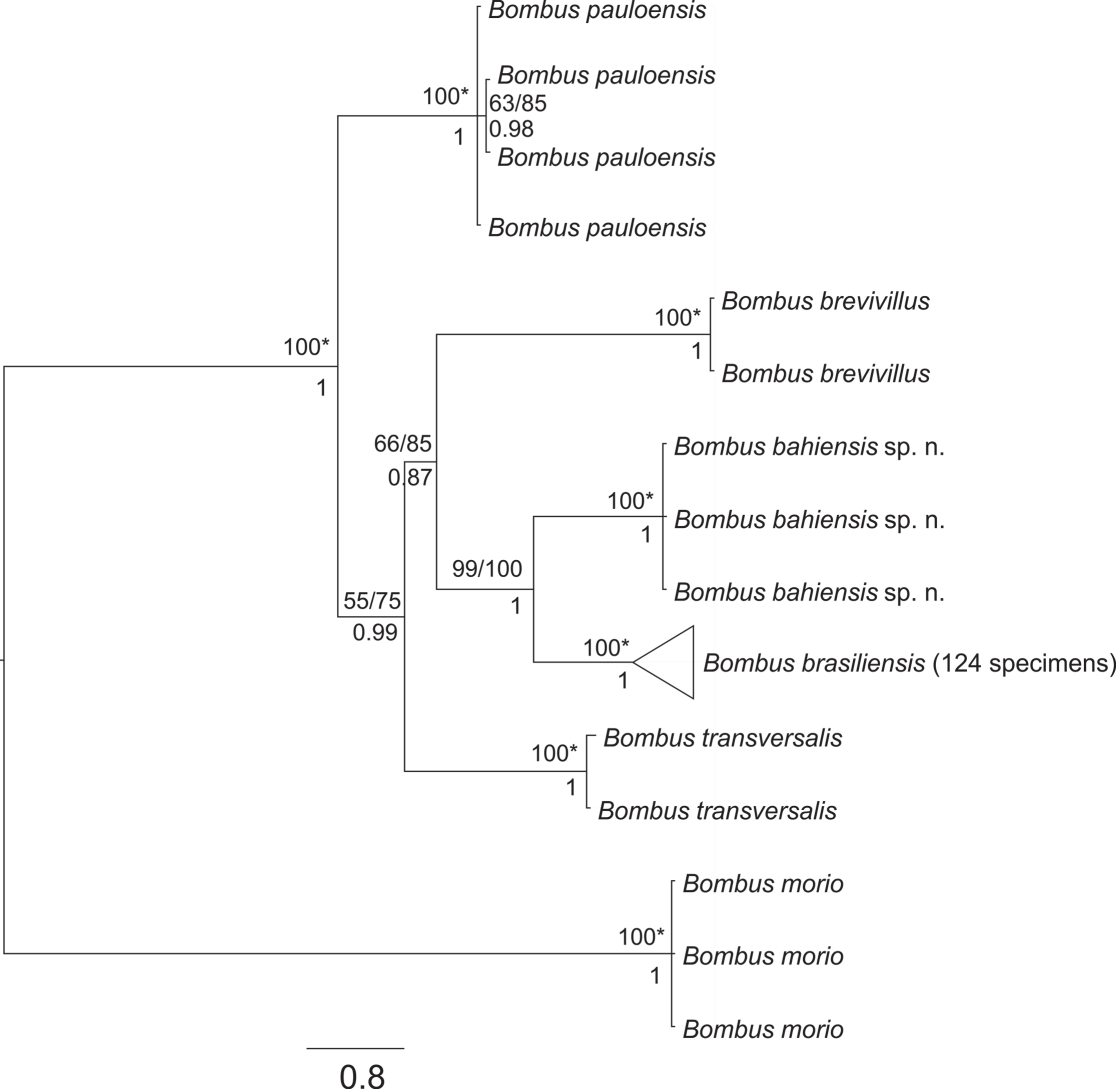
Phylogenetic relationships among Brazilian bumblebees obtained from concatenated phylogenetic analyses performed with CytB and COI sequences, using Bayesian inference and all specimens available for each species. Values shown under each branch are posterior probabilities. The values shown above each branch are bootstrap values represented here by Maximum Parsimony (MP)/ Maximum Likelihood (ML), because an identical topology was obtained with MP and ML. An analysis employing only unique haplotypes yields the same topology. “*” Represents the same values for MP and ML. *Bombus morio* (Swederus, 1787) was used as outgroup. The collapsed branch includes all *Bombus brasiliensis* haplotypes found on the haplotype network from Fig 2 and S1 Fig.

The phylogenetic analyses yielded the same single topology for Parsimony, Maximum Likelihood and Bayesian analysis (Fig 4). This tree was yielded after 1,102,282 rearrangements in the MP analyses and was 302-steps long. The average standard deviation of split frequencies after 20 million generations in the Bayesian analysis was of 0.002869, indicating convergence to a single subset of trees.

The phylogenetic analyses are congruent with the population analyses in suggesting that populations of *B*. *brasiliensis* isolated on the top of mountain ranges in southeastern Brazil belong to the same species as those in the Atlantic coast and in the lowlands of the Paraná-river valley (S1 Fig). They also show that specimens originally attributed to *B*. *brasiliensis* in the coastal forest of southern Bahia constitute a clade *(B*. *bahiensis* sp. n.), sister to the rest of the populations of *B*. *brasiliensis*.

### Morphology and taxonomic decisions

Examination of the external morphology of specimens attributed by the molecular analyses to *B*. *brasiliensis* and to *B*. *bahiensis* sp. n. revealed a character also allowing for their distinction: The pilosity of T1, which is entirely yellow in *B*. *brasiliensis* and mostly black in *B*. *bahiensis* (see key below). Furthermore, this character led to the recognition that two additional specimens from Conceição da Barra (northern Espírito Santo state) also belong in *B*. *Bahiensis* sp.n. (these latter specimens were too old and DNA extraction from them was not attempted). Considering the molecular and morphological evidence, the specimens from southern Bahia attributed to *B*. *brasiliensis* in our samples are considered here to be a species distinct from that containing the remaining populations of *B*. *brasiliensis*.

Two pieces of evidence indicate that the name *B*. *brasiliensis* should be applied to the species occurring along the coast of Rio de Janeiro and southwards: *a)* the holotype of *B*. *brasiliensis* has its first metasomal tergum covered with yellow pilosity (Fig 1B), as opposed to black, as in specimens from southern Bahia (Fig 1C and 1D); and *b)* the fact that this type specimen was probably collected in the vicinities of the city of Rio de Janeiro [23].

Moreover, two synonyms are recognized for *B*. *brasiliensis*: *B*. *venustus* Smith, 1861 and *B*. *brasiliensis* var. *palliventris* Friese, 1931 [23, 24], the former described from Teresópolis (on the top of the Serra do Mar mountain range, in the state of Rio de Janeiro—Fig 3) and the latter from Paraguay. The fact that populations sampled in Paraguay and on the mountaintops in southeastern Brazil showed to be conspecific with specimens from the southern Atlantic coast in the phylogenetic and population-genetic analyses, indicate that those names should continue to be considered as synonyms of *B*. *brasiliensis*. Taking all above in account, the populations occurring to the north of the Doce river, in the coastal forests of northern Espírito Santo and southern Bahia, are described below as a new species.

### Taxonomic treatment

#### *Bombus bahiensis* Santos Júnior & Silveira sp. n.

urn:lsid:zoobank.org:act:D4CB4F65-BA90-4E9B-BFCF-F420A9718C25

Fig 1C and 1D

**Diagnosis.** No single morphological character is known to be exclusive of *Bombus bahiensis* sp. n., which can be distinguished from most other Brazilian bumblebee species, except *B*. *brasiliensis*, for the mesosoma dorsally yellow, traversed by a black intertegular band, which extends laterally over the lower half of the mesepisternum (and generally reaching its ventral area), and the predominantly yellow metasoma traversed by a black band in T3; from *B*. *brasiliensis* it can be distinguished by the mostly black-haired T1, with yellow and partially-yellow hairs restricted to the mid-apical margin of the tergum. Only two species are potentially sympatric with *B*. *bahiensis* sp. n., *B*. *pauloensis* (melanic form) and *B*. *brevivillus*, which can be readily distinguished from *B*. *bahiensis* for their entirely black pilosity.

**Description (worker holotype).** *Measurements* (mm): approximate body length—16.2; head (length:width)– 4.5: 4.0; labrum (length: width)– 0.4:1.7; malar area (length × width)– 0.9:1.3; clypeocellar distance—1.9; interantennal distance—0.7; antennocular distance—0.6; ocellocular distance—0.6; interocellar distance—0.7; ocellar diameter—0.2; ocelloccipital distance—0.8; scape (length:diameter)– 2.1:0.3; length of F1 and F2–0.5, 0.3; forewing length—14.3; length of 2^nd^and 3^rd^ submarginal cells (measured on their posterior margins)– 1.7, 1.3.

*Structure*: labrum biconvex; disc of clypeus depressed; malar area slightly shorter than wide; interocellar distance larger than ocellorbital.

*Body color*: black, except reddish-brown on posterior tibia; wings dark brown, with light cupreous hue.

*Body surface*: clypeus coarsely punctate, except on disc, irregularly punctate with shiny interspaces; area between lateral ocelli and eye impunctate, smooth and shiny, except for a micro-punctate region on upper paraocular area, near upper inner-margin of compound eyes, which is narrower than mid-ocellar diameter, generally occupying one third of ocellorbital distance; glabrous area on disc of mesoscutum ill-defined, mostly restricted to region posterior to median mesoscutal line, lightly micro-reticulate but shiny; glabrous shiny area on inner surface of hind femur relatively narrow (less than half the width of the surface) occupying the apical three-fourths of the femoral length.

*Pubescence*: on head, black bristles intermixed with greyish plumose hairs, most abundant between antennal sockets; on mesosoma, long, fine hairs with long branches, light yellow, except for black intertegular band; on inner surface of hind tibia, fine, spatulate, the flat apical portion triangular, restricted to the very tip of each seta; on T1- T5 long and fine, with long branches, on T3 light yellow, on other terga, black; on T6 black and short; on sterna and legs, black and short.

*Variation*: the integument varies from black to reddish brown, especially on legs and metasoma; black hairs with light-yellow tips may occur on T1.

**Queen.** Similar to worker, except for the vertex much elevated above level of ocelli, as normally occurs in *Bombus* queens.

**Male.** Unknown.

**Holotype.** “Ilhéus BA; Brasil 18/11/2009; A. Nemésio”, “Euglossina da Hiléia Baiana, Campus UESC; 16724–47065”, “*Bombus bahiensis*; Santos Júnior & Silveira, sp. n.; HOLOTYPUS”. Collected in flight. Deposited at UFMG (accession number 16724–47065). The holotype lacks the right hind-leg, removed for DNA extraction.

**Paratypes.** “Euglossina da Hiléia Baiana, Campus UESC; 18339–52893”, “Ilhéus BA; Brasil 20/02/2010; A. Nemésio”, “*Bombus bahiensis*; Santos Júnior & Silveira sp. n.; PARATYPUS”; “Euglossina da Hiléia Baiana, Campus UESC; 18339–52894”, “Ilhéus BA; Brasil 20/02/2010; A. Nemésio”, “*Bombus bahiensis*; Santos Júnior & Silveira, sp. n.; PARATYPUS” (both workers, deposited at UFMG). “Conceição da Barra ES; Brasil 31/01/1969; C. Elias & T. Elias”, “*Bombus bahiensis*; Santos Júnior & Silveira, sp. n.; PARATYPUS” (two workers, deposited at DZUP).“Ilhéus BA; Brasil 2003; M. A. Costa”, “*Bombus bahiensis;* Santos Júnior & Silveira, sp. n.; PARATYPUS” (queen, deposited at DZUP).

**Etymology.** The name *bahiensis* refers both to the Brazilian state of Bahia, where the holotype and some of the paratypes were collected, and to its habitat, the Bahia Forest (see explanation below, under “distribution”).

**Distribution.** The species seems to be restricted to the so-called Bahia Forest, which is the especially luxuriant Atlantic Forest formation that covers southern Bahia and northern Espírito Santo states, in Brazil (Fig 3).

#### Key to females of Brazilian species of *Bombus* Latreille, 1802

(Modified from [23])

**-** Pilosity entirely black, except for apically-pale hairs on ventral parts of body ........ **2**
**-** Pilosity at least partially yellow on mesosoma, metasoma or both, forming bands or entirely covering one or more terga .......................................................... **4**
**-** Malar area longer than its apical width; posterior glabrous area on disc of mesoscutum well delimited, micro-reticulate and dull ....***B*. *morio* (Swederus, 1787)**
**-** Malar area shorter than its apical width; posterior glabrous area on disc of mesoscutum poorly or well delimited but never micro-reticulate and shiny ........... **3**
**-** Micro-punctate region on upper paraocular area, near upper inner-margin of compound eyes wide, as wide as or wider than mid-ocellar diameter, occupying half of ocellorbital distance; pilosity relatively dense, velvety, especially dorsally on mesoscutum of queens ....... . . .. . . .. . .. . ....... *B*. *brevivillus* Franklin, 1913*
**-** Micro-punctate region on upper paraocular area, near upper inner-margin of compound eyes narrow, narrower than mid-ocellar diameter, generally occupying one third of ocellorbital distance; pilosity looser, not velvety ................................... ......................................................... ***pauloensis* Friese, 1913** (black form)
**-** Pilosity of pronotum, mesoscutum and scutellum light-yellow to yellowish-brown, without black intertegular hair-band; pilosity on T4-T6 ferruginous ................................... ......................................................... ***B*. *bellicosus* Smith, 1879**
**-** Yellow pilosity on pronotum, mesoscutum and scutellum relatively well developed, but always with an intertegular black band; T3 generally with yellow hair band; T4-T6 black-haired ................................................................................. **5**
**-** Yellow pilosity on mesosoma never totally covering meso and metepisterna, which are black haired on lower half, including venter ........................................... **6**
**-** Yellow pilosity entirely covering meso and metepisterna, frequently reaching venter of mesosoma ........................................ . ................................ . ............ **7**
**-** Posterior glabrous area on disc of mesoscutum ill-defined, with sparse punctures reaching its middle; pilosity on pronotum, scutellum and T3 deep yellow, black intertegular band narrower than pronotal band ........ ***B*. *transversalis* (Olivier, 1789)**
**-** Posterior glabrous area on disc of mesoscutum well defined, with no punctures on its middle; yellow pilosity entirely light; intertegular hair band wider than pronotal band ........................................... ***B*. *pauloensis* Friese, 1913** (yellow-banded form)
**-** T1 covered with yellow pilosity .........................***B*. *brasiliensis* Lepeletier, 1836**
**-** T1 covered with black pilosity, frequently intermingled with yellow or partially yellow hairs mid-apically ................ ***B*. *bahiensis* Santos Júnior & Silveira sp. n.**

* Probably a compound species (see text).

## Discussion

### Variation discontinuity, Phylogeny and Species Status

The genetic distance between *B*. *bahiensis* sp. n. and *B*. *brasiliensis* was relatively small, and this may raise the suspicion that they might in fact be a single species. However, comparable small divergences have been commonly estimated for several species-pairs believed to have diverged recently, in other bee taxa. For example, divergences found for COI-sequences by Dick et al. [44] and Nemésio et al. [45] between species pairs of orchid-bee (Euglossina) in South America were all below 2%, while Gibbs [2] found only a 3.06% average COI-sequence divergence among five species of *Lasioglossum* previously misinterpreted as a single species (with the lowest recorded value of 1.7%*)*. In *Bombus*, such small genetic distances were found, also, among cryptic species in northern Europe ([5, 10], see also Table 5 in [12]). Moreover, although the average genetic distance between *B*. *brasiliensis* and *B*. *bahiensis* sp. n. was the smallest recorded between pairs of Brazilian species of *Bombus* in this study, it was higher, for example, than those obtained for seven other pairs of undisputed species in Asia, Europe, and South America (S3 Table). The fact that the smallest distance between a specimen of *B*. *brasiliensis* and one of *B*. *bahiensis* is 2.5 times larger than the largest distance between any two specimens of *B*. *brasiliensis* is an additional support for a genetic gap between *B*. *bahiensis* and *B*. *brasiliensis*.

Traditionally, discontinuity in variation among populations has been considered, both in morphological and molecular-based studies, as evidence that independently-evolving lineages are involved (for example [29]). In the barcoding context, average intra-population divergences much smaller than the inter-population divergences (the so called barcode gap—[46]) is considered evidence that they are cohesive sets of populations, isolated from each other. Much has been discussed in the literature about the reality and convenient thresholds of such a gap (for example [46]). However, there is some agreement that one is safe in recognizing different species when the barcode gap between them reaches the order of one magnitude, as in the case of *B*. *brasiliensis* and *B*. *bahiensis* sp. n. A problem here is that large intra-specific divergence could not be expected for *B*. *bahiensis* sp. n., considering that it is represented by a small sample from a single site. However, the situation could be looked at from the other way around: *B*. *brasiliensis* from sites more than 1500 km apart along the latitudinal gradient or from isolated populations in areas differing more than 1500 m in elevation did not show divergences as large as that found between populations of *B*. *bahiensis* sp. n. and those of *B*. *brasiliensis* about 240 Km apart in lowland evergreen forests.

The fact that *B*. *brasiliensis* and *B*. *bahiensis* sp. n. are reciprocally monophyletic also supports their recognition as separate species (for example [47]), as it suggests that each of them is a mutually-independent evolutionary lineage. It could be argued that the phylogenetic hypothesis produced here, being based on two linked genes (maternally-inherited mtDNA), may merely indicate a phylogeny for those genes, which may be different from the correct phylogeny of the species involved (for example [48]). Nevertheless, as pointed out by Williams et al. [49], this problem has not been detected for the use of COI in bumblebees and, moreover, *mt*DNA may actually be more suitable for species delimitation than other molecular and morphological data [50].

### Biogeographic considerations

Before the arrival of the Portuguese settlers, the presumed geographic range of *B*. *bahiensis* sp. n. was covered by an especially luxurious evergreen forest (from now on, the Bahia forest), coinciding with an important area of endemism for many plant and animal taxa, including insects [51], and including several recently-described endemic species of orchid bees (Apidae: Euglossina) (for example [52–56]). Intra- and interspecific disjunctions of populations or sister-species involving this area and other areas in the Amazonian Forest or to the south in the Atlantic Forest also have been recorded for several taxa (for example [57–59]).

A closer look to the distributions of *B*. *brasiliensis* and *B*. *bahiensis* sp. n. shows a 240-km gap between the northernmost known record of the species in the coastal lowlands of Rio de Janeiro state (in the municipality of São Francisco de Itabapoana, see Fig 3 and S1 Table), and its southernmost record in the lowlands of Espírito Santo state (municipality of Linhares; [23]—see comments on this population below) (Fig 3). *Bombus brasiliensis* does occur in southeastern Brazil, in latitudes between those of Linhares and São Francisco de Itabapoana [23, 28, 60], but all these records (and additional localities represented in the UFMG collection) refer to cloud forests (or their immediate vicinities) on the top of mountain chains, further inland. These mountaintop records represent populations which are isolated from those near the coast (Fig 3) by semi-deciduous forests in areas under climates with relatively long dry seasons (4–6 months)—the same kind of environment found in the gap between the humid northern area occupied by *B*. *bahiensis* sp. n. and the humid southern area occupied by *B*. *brasiliensis* (compare, for example, the map in Fig 3 with that of the Atlantic Forest vegetation types presented by Carnaval et al. [61] in their supplementary material’ Figure S1).

Close relationship between elements of the Bahia forest and the Amazonian Forest biotas has been pointed out for organisms such as birds (for example [62]) and also for orchid bees, with several pairs of presumed sister species of the latter occurring disjunctly in the two phytogeographic domains (for example [54, 55]). This may suggest that *B*. *bahiensis* sp. n. could be sister to the only species in the genus restricted to Amazonia, *B*. *transversalis* [23]. However, this hypothesis is not supported by any of the results obtained here.

The presumed range of *B*. *bahiensis* sp. n. also falls within a forest refuge that existed in the Atlantic Forest domain during cold, dry periods of the Pleistocene, the so-called Bahia refuge, which was predicted by climatic models and validated by paleopalynological data (for example [61, 63,64]). In the Bahia refuge, limited in the south by the Doce river, in Espírito Santo, and extending northward into southern Bahia, evergreen forests persisted even in the driest periods of the Pleistocene. Carnaval et al. [64] predicted that surveys in this area would still reveal undescribed species and cryptic lineages. If Hines [65] is right in her estimate that the closest common ancestor of *B*. *brasiliensis*, *B*. *transversalis* and *B*. *pauloensis* existed at about 2 Mya, then the most recent common ancestor of *B*. *brasiliensis* and *B*. *bahiensis* sp. n. lived in the Pleistocene, and this would be consistent with the hypothesis that the isolation of part of its populations in the Bahia refuge during that period could be the vicariance event responsible for its genetic divergence. The close association of *B*. *brasiliensis* and *B*. *bahiensis* sp. n. with evergreen forests in southeastern Brazil suggests that interbreeding of their populations may have been prevented by their inability to settle in lowland semi-deciduous forests, which occur under climates with relatively long dry seasons and that reaches the coast between southern Espírito Santo and northern Rio de Janeiro states. It should be noted, however, that this hypothesis is weakened by the occurrence of *B*. *brasiliensis* in areas dominated by climates with relatively long dry seasons and covered with semideciduous forests along the Paraná River valley, westward into Paraguay and central Brazil (Fig 3). A closer look at the habitats used by this species in that region is necessary for a better understanding of this question.

### Morphological distinction and geographic range

*Bombus* is a relatively monotonous genus, as far as morphological characters are concerned (for example [1]), and this makes them frequently difficult to distinguish, especially among closely-related species (for example [5–11, 49]. Thus, it is not surprising that not a single exclusive autoapomorphy of *B*. *bahiensis* sp. n. could be found, and that the only morphological character distinguishing it from its presumed sister species, the black-haired T1, is a plesiomorphic trait or, at most, a homoplastic apomorphy, also found in all other Brazilian species but *B*. *brasiliensis* (for example [23]).

Moure & Sakagami [23] noticed that specimens identified by them as *B*. *brasiliensis* from Linhares, by the northern margin of the Doce river, in Espírito Santo state (shown by a grey triangle with a central black dot in Fig 3) had their first metasomal tergum completely covered by black pilosity. They interpreted this population as a melanic variety of *B*. *brasiliensis*. These specimens quite probably belong to *B*. *bahiensis* sp. n. and may represent the southernmost population of the species, since the Doce river is the southern limit for the evergreen forests of the Bahia refuge and the Bahia forest (although some authors, as Silva & Casteleti [51] and Ribeiro et al. [66] consider the Bahia forest to extend further south in Espírito Santo).

### Conservation Status

Local or regional decline or extirpation of bumblebee populations has been reported in the northern hemisphere (for example [49, 67–70]) and in Brazil [71]. It seems that the small available sample of *B*. *bahiensis* sp. n. reflects a low abundance in nature and not a small sampling effort in its habitat. The bee fauna of northern Espírito Santo was intensively sampled by C. Elias, a collector for the DZOL collection in Curitiba in the 1960’s and early 1970’s, and the only specimens of the species collected among thousands of bee specimens, apparently are those few listed in the “Taxonomic treatment”. Moreover, two expeditions were set to southern Bahia and northern Espírito Santo to search for the species, during the development of this project (January and June/July, 2014), but resulted unsuccessful. With this in mind, two facts should be considered concerning the conservation of this new species: 1) its presumed geographic range is the smallest among all Brazilian *Bombus* species; and 2) its natural habitat is under heavy anthropic impact—the rain forest that originally covered about 86% of the region, now covers only between 12% and 17% of it [51, 66]—and continues to be fragmented. Moreover, bees in general and bumblebees specifically may be more susceptible to fragmentation than other organisms, due to issues related to effective population size, social behavior, nesting, sex determination mechanisms etc. (see discussions in [72, 68]). Thus, *B*. *bahiensis* sp. n. may actually be an endangered species and efforts should be made to map the remaining populations of the species and their abundances, better defining its current geographic range and conservation status.

## Supporting Information

S1 Fig Phylogenetic relationships among *Bombus brasiliensis* obtained from concatenated phylogenetic analyses performed with CytB and COI sequences, using Bayesian inference. Values shown under each branch are posterior probabilities.
(TIF)

S1 Table Specimens sequenced for the genetic analyses with their geographic origins. Universidade Federal de Minas Gerais—UFMG IHY; Universidade Federal de Santa Catarina—UFSC; Universidade Federal do Norte Fluminense—UENF; Universidade Federal de Ouro Preto—UFOP; Faculdade de Filosofia, Ciências e Letras de Ribeirão Preto—Universidade de São Paulo—USP: FFCLRP; Universidade de São Paulo—USP/SP.
(DOCX)

S2 Table Protocol of the purification of Polyethylene Glycol 20% (PEG 20%) for elimination of bands <300–400 bp. (DOCX)

S3 Table Genetic distances for COI sequences (%) between bumblebee-species. **The model used was the Kimura 2-parameter.** Bees currently considered as *B*. *brevivillus* belong in two distinct species, identified below as *B*. *brevivillus* (1) and *B*. *brevivillus* (2). AD = Average interspecific distance ± standard deviation; MiD = minimum interespecific distance ± standard deviation.
(DOCX)
