# Supplementary figures and images for: Hitting an Unintended Target: Phylogeography of Bombus brasiliensis Lepeletier, 1836 and the First New Brazilian Bumblebee Species in a Century (Hymenoptera: Apidae)
Source: PLoS One. 2015 May 20;10(5):e0125847. doi: 10.1371/journal.pone.0125847 (PMC4438978; doi:10.1371/journal.pone.0125847)

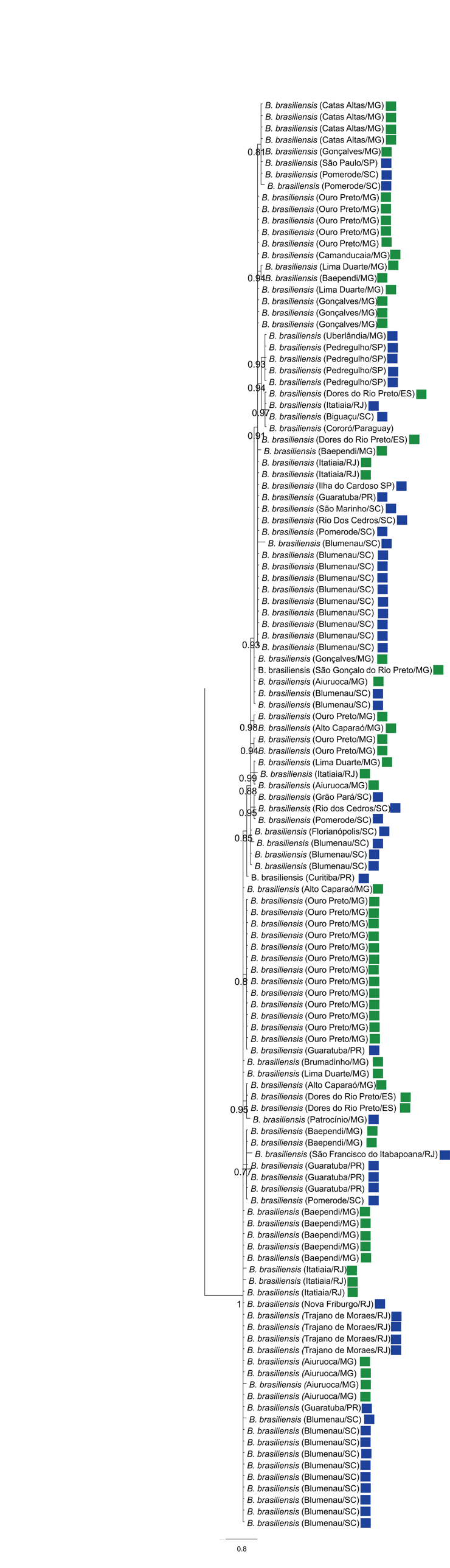

Supplement: S1 Fig — Values shown under each branch are posterior probabilities. (TIF) [file pone.0125847.s001.tif]
