# Supplementary material for: Hitting an Unintended Target: Phylogeography of Bombus brasiliensis Lepeletier, 1836 and the First New Brazilian Bumblebee Species in a Century (Hymenoptera: Apidae)
Source: PLoS One. 2015 May 20;10(5):e0125847. doi: 10.1371/journal.pone.0125847 (PMC4438978; doi:10.1371/journal.pone.0125847)
Supplement: S1 Table — Universidade Federal de Minas Gerais—UFMG IHY; Universidade Federal de Santa Catarina—UFSC; Universidade Federal do Norte Fluminense—UENF; Universidade Federal de Ouro Preto—UFOP; Faculdade de Filosofia, Ciências e Letras de Ribeirão Preto—Universidade de São Paulo—USP: FFCLRP; Universidade de São Paulo—USP/SP. (DOCX) [file pone.0125847.s002.docx]

**Table S1. Specimens sequenced for the genetic analyses with their geographic origins.** Universidade Federal de Minas Gerais – UFMG IHY; Universidade Federal de Santa Catarina – UFSC; Universidade Federal do Norte Fluminense – UENF; Universidade Federal de Ouro Preto – UFOP; Faculdade de Filosofia, Ciências e Letras de Ribeirão Preto – Universidade de São Paulo – USP: FFCLRP; Universidade de São Paulo – USP/SP.

| Organism | GenBank Assession Number | | Museum ID | Municipality | State/Department/Province | Country |
| --- | --- | --- | --- | --- | --- | --- |
|  | COI | CytB |  |  |  |  |
| *Bombus pauloensis* Friese, 1913 | KJ848786 |  | UFMG IHY 77202 | Salinas | Minas Gerais | Brazil |
| *Bombus pauloensis* Friese, 1913 | KJ848787 | KJ848954 | UFMG IHY 931 | Belo Horizonte | Minas Gerais | Brazil |
| *Bombus pauloensis* Friese, 1913 | KJ848788 | KJ848955 | UFMG IHY 33893 | Ibirité | Minas Gerais | Brazil |
| *Bombus brasiliensis* Lepeletier, 1836 | KJ848789 | KJ848956 | UFSC CJS2917 | Pomerode | Santa Catarina | Brazil |
| *Bombus brasiliensis* Lepeletier, 1836 | KJ848790 | KJ848957 | UENF | São Francisco do Itabapoana | Rio de Janeiro | Brazil |
| *Bombus brasiliensis* Lepeletier, 1836 | KJ848791 | KJ848958 | UFSC CJS2933 | Grão Pará | Santa Catarina | Brazil |
| *Bombus brasiliensis* Lepeletier, 1836 | KJ848792 |  | UFSC CJS2935 | Florianópolis | Santa Catarina | Brazil |
| *Bombus brasiliensis* Lepeletier, 1836 | KJ848793 | KJ848959 | UFSC CJS2965 | Pomerode | Santa Catarina | Brazil |
| *Bombus brasiliensis* Lepeletier, 1836 | KJ848794 | KJ848960 | UFSC CJS4060 | Florianópolis | Santa Catarina | Brazil |
| *Bombus brasiliensis* Lepeletier, 1836 | KJ848795 | KJ848961 | UFSC CJS5233 | Pomerode | Santa Catarina | Brazil |
| *Bombus brasiliensis* Lepeletier, 1836 | KJ848796 | KJ848962 | UFSC CJS5234 | Rio dos Cedros | Santa Catarina | Brazil |
| *Bombus brasiliensis* Lepeletier, 1836 | KJ848797 | KJ848963 | UFSC CJS5237 | Pomerode | Santa Catarina | Brazil |
| *Bombus brasiliensis* Lepeletier, 1836 | KJ848798 | KJ848964 | UFSC CJS5238 | Pomerode | Santa Catarina | Brazil |
| *Bombus brasiliensis* Lepeletier, 1836 | KJ848799 | KJ848965 | UFSC CJS5239 | Rio dos Cedros | Santa Catarina | Brazil |
| *Bombus morio* (Swederus, 1787) | KJ848800 | KJ848966 | UFMG IHY 33667 | Brasília | Distrito Federal | Brazil |
| *Bombus brasiliensis* Lepeletier, 1836 | KJ848801 | KJ848967 | UFSC CJS9045 | São Martinho | Santa Catarina | Brazil |
| *Bombus morio* (Swederus, 1787) | KJ848802 | KJ848968 | UFMG IHY 39038 | Aiuruoca | Minas Gerais | Brazil |
| *Bombus brasiliensis* Lepeletier, 1836 | KJ848803 | KJ848969 | UENF | Trajano de Moraes | Rio de Janeiro | Brazil |
| *Bombus brasiliensis* Lepeletier, 1836 | KJ848804 | KJ848970 | UENF | Trajano de Moraes | Rio de Janeiro | Brazil |
| *Bombus brasiliensis* Lepeletier, 1836 | KJ848805 | KJ848971 | UENF | Trajano de Moraes | Rio de Janeiro | Brazil |
| *Bombus brasiliensis* Lepeletier, 1836 | KJ848806 | KJ848972 | UENF | Trajano de Moraes | Rio de Janeiro | Brazil |
| *Bombus brasiliensis* Lepeletier, 1836 | KJ848807 | KJ848973 | UENF | Catas Altas | Minas Gerais | Brazil |
| *Bombus brasiliensis* Lepeletier, 1836 | KJ848808 | KJ848974 | UFMG IHY 1218400 | Pedregulho | São Paulo | Brazil |
| *Bombus brasiliensis* Lepeletier, 1836 | KJ848809 | KJ848975 | UFMG IHY 1218401 | Pedregulho | São Paulo | Brazil |
| *Bombus brasiliensis* Lepeletier, 1836 | KJ848810 | KJ848976 | UFMG IHY 1218402 | Pedregulho | São Paulo | Brazil |
| *Bombus brasiliensis* Lepeletier, 1836 | KJ848811 | KJ848977 | UFMG IHY 1218403 | Pedregulho | São Paulo | Brazil |
| *Bombus brasiliensis* Lepeletier, 1836 | KJ848812 | KJ848978 | UFOP | Ouro Preto | Minas Gerais | Brazil |
| *Bombus morio* (Swederus, 1787) | KJ848813 | KJ848979 | UFMG IHY | Catalão | Goiás | Brazil |
| *Bombus brasiliensis* Lepeletier, 1836 | KJ848814 | KJ848980 | UFOP | Ouro Preto | Minas Gerais | Brazil |
| *Bombus brasiliensis* Lepeletier, 1836 | KJ848815 | KJ848981 | USP: FFCLRP | Blumenau | Santa Catarina | Brazil |
| *Bombus brasiliensis* Lepeletier, 1836 | KJ848816 | KJ848982 | USP: FFCLRP | Blumenau | Santa Catarina | Brazil |
| *Bombus brasiliensis* Lepeletier, 1836 | KJ848817 | KJ848983 | USP: FFCLRP | Blumenau | Santa Catarina | Brazil |
| *Bombus brasiliensis* Lepeletier, 1836 | KJ848818 | KJ848984 | USP: FFCLRP | Blumenau | Santa Catarina | Brazil |
| *Bombus brasiliensis* Lepeletier, 1836 | KJ848819 | KJ848985 | USP: FFCLRP | Blumenau | Santa Catarina | Brazil |
| *Bombus brasiliensis* Lepeletier, 1836 | KJ848820 | KJ848986 | USP: FFCLRP | Blumenau | Santa Catarina | Brazil |
| *Bombus brasiliensis* Lepeletier, 1836 | KJ848821 | KJ848987 | USP: FFCLRP | Blumenau | Santa Catarina | Brazil |
| *Bombus brasiliensis* Lepeletier, 1836 | KJ848786 | KJ848988 | USP: FFCLRP | Blumenau | Santa Catarina | Brazil |
| *Bombus bahiensis* sp. n. | KJ848823 | KJ848989 | UFMG IHY 47065 | Ilhéus | Bahia | Brazil |
| *Bombus brasiliensis* Lepeletier, 1836 | KJ848824 | KJ848990 | USP: FFCLRP | Blumenau | Santa Catarina | Brazil |
| *Bombus brasiliensis* Lepeletier, 1836 | KJ848825 | KJ848991 | USP: FFCLRP | Blumenau | Santa Catarina | Brazil |
| *Bombus brasiliensis* Lepeletier, 1836 | KJ848826 | KJ848992 | USP: FFCLRP | Blumenau | Santa Catarina | Brazil |
| *Bombus brasiliensis* Lepeletier, 1836 | KJ848827 | KJ848993 | USP: FFCLRP | Blumenau | Santa Catarina | Brazil |
| *Bombus brasiliensis* Lepeletier, 1836 | KJ848828 | KJ848994 | USP: FFCLRP | Blumenau | Santa Catarina | Brazil |
| *Bombus brasiliensis* Lepeletier, 1836 | KJ848829 | KJ848995 | USP: FFCLRP | Blumenau | Santa Catarina | Brazil |
| *Bombus brasiliensis* Lepeletier, 1836 | KJ848830 | KJ848996 | USP: FFCLRP | Blumenau | Santa Catarina | Brazil |
| *Bombus brasiliensis* Lepeletier, 1836 | KJ848831 | KJ848997 | USP: FFCLRP | Blumenau | Santa Catarina | Brazil |
| *Bombus brasiliensis* Lepeletier, 1836 | KJ848832 | KJ848998 | USP: FFCLRP | Blumenau | Santa Catarina | Brazil |
| *Bombus brasiliensis* Lepeletier, 1836 | KJ848833 | KJ848999 | USP: FFCLRP | Blumenau | Santa Catarina | Brazil |
| *Bombus brasiliensis* Lepeletier, 1836 | KJ848834 | KJ849000 | USP: FFCLRP | Blumenau | Santa Catarina | Brazil |
| *Bombus brasiliensis* Lepeletier, 1836 | KJ848835 | KJ849001 | USP: FFCLRP | Blumenau | Santa Catarina | Brazil |
| *Bombus brasiliensis* Lepeletier, 1836 | KJ848836 | KJ849002 | USP: FFCLRP | Blumenau | Santa Catarina | Brazil |
| *Bombus brasiliensis* Lepeletier, 1836 | KJ848837 | KJ849003 | USP: FFCLRP | Blumenau | Santa Catarina | Brazil |
| *Bombus brasiliensis* Lepeletier, 1836 | KJ848838 | KJ849004 | USP: FFCLRP | Blumenau | Santa Catarina | Brazil |
| *Bombus brasiliensis* Lepeletier, 1836 | KJ848839 | KJ849005 | USP: FFCLRP | Blumenau | Santa Catarina | Brazil |
| *Bombus brasiliensis* Lepeletier, 1836 | KJ848840 | KJ849006 | USP: FFCLRP | Blumenau | Santa Catarina | Brazil |
| *Bombus brasiliensis* Lepeletier, 1836 | KJ848841 | KJ849007 | UFMG IHY | Nova Friburgo | Rio de Janeiro | Brazil |
| *Bombus brasiliensis* Lepeletier, 1836 | KJ848842 |  | UFOP | Ouro Preto | Minas Gerais | Brazil |
| *Bombus brasiliensis* Lepeletier, 1836 | KJ848843 |  | UFOP | Ouro Preto | Minas Gerais | Brazil |
| *Bombus brasiliensis* Lepeletier, 1836 | KJ848844 | KJ849008 | UFOP | Ouro Preto | Minas Gerais | Brazil |
| *Bombus brasiliensis* Lepeletier, 1836 | KJ848845 | KJ849009 | UFOP | Ouro Preto | Minas Gerais | Brazil |
| *Bombus brasiliensis* Lepeletier, 1836 | KJ848846 | KJ849010 | UFOP | Ouro Preto | Minas Gerais | Brazil |
| *Bombus brasiliensis* Lepeletier, 1836 | KJ848847 | KJ849011 | UFOP | Ouro Preto | Minas Gerais | Brazil |
| *Bombus brasiliensis* Lepeletier, 1836 | KJ848848 |  | UFOP | Ouro Preto | Minas Gerais | Brazil |
| *Bombus brasiliensis* Lepeletier, 1836 | KJ848849 | KJ849012 | UFOP | Ouro Preto | Minas Gerais | Brazil |
| *Bombus brasiliensis* Lepeletier, 1836 | KJ848850 | KJ849013 | UFOP | Ouro Preto | Minas Gerais | Brazil |
| *Bombus brasiliensis* Lepeletier, 1836 | KJ848851 |  | UFOP | Ouro Preto | Minas Gerais | Brazil |
| *Bombus brasiliensis* Lepeletier, 1836 | KJ848852 |  | UFOP | Ouro Preto | Minas Gerais | Brazil |
| *Bombus brasiliensis* Lepeletier, 1836 | KJ848853 |  | UFOP | Ouro Preto | Minas Gerais | Brazil |
| *Bombus brasiliensis* Lepeletier, 1836 | KJ848854 | KJ849014 | UFMG IHY 8514 | Catas Altas | Minas Gerais | Brazil |
| *Bombus brasiliensis* Lepeletier, 1836 | KJ848855 | KJ849015 | UFOP | Ouro Preto | Minas Gerais | Brazil |
| *Bombus brasiliensis* Lepeletier, 1836 | KJ848856 | KJ849016 | UFOP | Ouro Preto | Minas Gerais | Brazil |
| *Bombus brasiliensis* Lepeletier, 1836 | KJ848857 | KJ849017 | UFOP | Ouro Preto | Minas Gerais | Brazil |
| *Bombus brasiliensis* Lepeletier, 1836 | KJ848858 | KJ849018 | UFOP | Ouro Preto | Minas Gerais | Brazil |
| *Bombus brasiliensis* Lepeletier, 1836 | KJ848859 |  | UFOP | Ouro Preto | Minas Gerais | Brazil |
| *Bombus brasiliensis* Lepeletier, 1836 | KJ848860 | KJ849019 | UFOP | Ouro Preto | Minas Gerais | Brazil |
| *Bombus brasiliensis* Lepeletier, 1836 | KJ848861 | KJ849020 | UFOP | Ouro Preto | Minas Gerais | Brazil |
| *Bombus brasiliensis* Lepeletier, 1836 | KJ848862 | KJ849021 | UFOP | Ouro Preto | Minas Gerais | Brazil |
| *Bombus brasiliensis* Lepeletier, 1836 | KJ848863 | KJ849022 | UFOP | Ouro Preto | Minas Gerais | Brazil |
| *Bombus brasiliensis* Lepeletier, 1836 | KJ848864 |  | UFOP | Ouro Preto | Minas Gerais | Brazil |
| *Bombus brasiliensis* Lepeletier, 1836 | KJ848865 |  | UFMG IHY 16984 | Catas Altas | Minas Gerais | Brazil |
| *Bombus brasiliensis* Lepeletier, 1836 | KJ848866 |  | UFOP | Ouro Preto | Minas Gerais | Brazil |
| *Bombus brasiliensis* Lepeletier, 1836 | KJ848867 |  | UFOP | Ouro Preto | Minas Gerais | Brazil |
| *Bombus brasiliensis* Lepeletier, 1836 | KJ848868 |  | UFOP | Ouro Preto | Minas Gerais | Brazil |
| *Bombus brasiliensis* Lepeletier, 1836 | KJ848869 | KJ849023 | UFOP | Ouro Preto | Minas Gerais | Brazil |
| *Bombus brasiliensis* Lepeletier, 1836 | KJ848870 | KJ849024 | USP/SP | São Paulo | São Paulo | Brazil |
| *Bombus brasiliensis* Lepeletier, 1836 | KJ848871 | KJ849025 | USP/SP | Ilha do Cardoso | São Paulo | Brazil |
| *Bombus brasiliensis* Lepeletier, 1836 | KJ848872 | KJ849026 | USP/SP | Biguaçu | Santa Catarina | Brazil |
| *Bombus brasiliensis* Lepeletier, 1836 | KJ848873 |  | USP/SP | Biguaçu | Santa Catarina | Brazil |
| *Bombus brevivillus* Franklin, 1913 | KJ848874 | KJ849027 | UFMG IHY 1200916 | Prata | Paraíba | Brazil |
| *Bombus brasiliensis* Lepeletier, 1836 | KJ848875 |  | UFMG IHY 21524 | Catas Altas | Minas Gerais | Brazil |
| *Bombus brevivillus* Franklin, 1913 | KJ848876 | KJ849028 | UFMG IHY 1202754 | Remígio | Paraíba | Brazil |
| *Bombus pauloensis* Friese, 1913 | KJ848877 |  | UFMG IHY 19052 | Belo Horizonte | Minas Gerais | Brazil |
| *Bombus brasiliensis* Lepeletier, 1836 | KJ848878 | KJ849029 | UFMG IHY 22050 | Alto Caparaó | Minas Gerais | Brazil |
| *Bombus brasiliensis* Lepeletier, 1836 | KJ848879 |  | UFMG IHY 25936 | Brumadinho | Minas Gerais | Brazil |
| *Bombus brasiliensis* Lepeletier, 1836 | KJ848880 | KJ849030 | UFMG IHY 27247 | Ouro Preto | Minas Gerais | Brazil |
| *Bombus brasiliensis* Lepeletier, 1836 | KJ848881 |  | UFMG IHY 23059 | Curitiba | Paraná | Brazil |
| *Bombus brasiliensis* Lepeletier, 1836 | KJ848882 | KJ849031 | UFMG IHY 23061 | Curitiba | Paraná | Brazil |
| *Bombus brasiliensis* Lepeletier, 1836 | KJ848883 | KJ849032 | UFMG IHY 31382 | Ouro Preto | Minas Gerais | Brazil |
| *Bombus brasiliensis* Lepeletier, 1836 | KJ848884 | KJ849033 | UFMG IHY 39048 | Aiuruoca | Minas Gerais | Brazil |
| *Bombus brasiliensis* Lepeletier, 1836 | KJ848885 | KJ849034 | UFMG IHY 17568 | Catas Altas | Minas Gerais | Brazil |
| *Bombus brasiliensis* Lepeletier, 1836 | KJ848886 | KJ849035 | UFMG IHY 31481 | São Gonçalo do Rio Preto | Minas Gerais | Brazil |
| *Bombus brasiliensis* Lepeletier, 1836 | KJ848887 | KJ849036 | UFMG IHY 34105 | Ouro Preto | Minas Gerais | Brazil |
| *Bombus morio* (Swederus, 1787) | KJ848888 |  | UFMG IHY 30978 | São Gonçalo do Rio Preto | Minas Gerais | Brazil |
| *Bombus brasiliensis* Lepeletier, 1836 | KJ848889 | KJ849037 | UFMG IHY 34082 | Brumadinho | Minas Gerais | Brazil |
| *Bombus brasiliensis* Lepeletier, 1836 | KJ848890 | KJ849038 | UFMG IHY 33248 | Alto Caparaó | Minas Gerais | Brazil |
| *Bombus brasiliensis* Lepeletier, 1836 | KJ848891 |  | UFMG IHY 32200 | Camanducaia | Minas Gerais | Brazil |
| *Bombus brasiliensis* Lepeletier, 1836 | KJ848892 | KJ849039 | UFMG IHY 32217 | Camanducaia | Minas Gerais | Brazil |
| *Bombus brasiliensis* Lepeletier, 1836 | KJ848893 | KJ849040 | UFMG IHY 37805 | Itatiaia | Rio de Janeiro | Brazil |
| *Bombus brasiliensis* Lepeletier, 1836 | KJ848894 | KJ849041 | UFMG IHY 37816 | Itatiaia | Rio de Janeiro | Brazil |
| *Bombus brasiliensis* Lepeletier, 1836 | KJ848895 | KJ849042 | UFMG IHY 37833 | Itatiaia | Rio de Janeiro | Brazil |
| *Bombus brasiliensis* Lepeletier, 1836 | KJ848896 | KJ849043 | UFMG IHY 37985 | Lima Duarte | Minas Gerais | Brazil |
| *Bombus brasiliensis* Lepeletier, 1836 | KJ848897 | KJ849044 | UFMG IHY 37992 | Lima Duarte | Minas Gerais | Brazil |
| *Bombus brasiliensis* Lepeletier, 1836 | KJ848898 | KJ849045 | UFMG IHY 37993 | Lima Duarte | Minas Gerais | Brazil |
| *Bombus pauloensis* Friese, 1913 | KJ848899 |  | UFMG IHY 39784 | Gonçalves | Minas Gerais | Brazil |
| *Bombus brasiliensis* Lepeletier, 1836 | KJ848900 | KJ849046 | UFMG IHY 38336 | Alto Caparaó | Minas Gerais | Brazil |
| *Bombus brasiliensis* Lepeletier, 1836 | KJ848901 | KJ849047 | UFMG IHY 37853 | Itatiaia | Rio de Janeiro | Brazil |
| *Bombus brasiliensis* Lepeletier, 1836 | KJ848902 | KJ849048 | UFMG IHY 37860 | Itatiaia | Rio de Janeiro | Brazil |
| *Bombus brasiliensis* Lepeletier, 1836 | KJ848903 | KJ849049 | UFMG IHY 37867 | Itatiaia | Rio de Janeiro | Brazil |
| *Bombus brasiliensis* Lepeletier, 1836 | KJ848904 | KJ849050 | UFMG IHY 37883 | Itatiaia | Rio de Janeiro | Brazil |
| *Bombus brasiliensis* Lepeletier, 1836 | KJ848905 | KJ849051 | UFMG IHY 38011 | Lima Duarte | Minas Gerais | Brazil |
| *Bombus brasiliensis* Lepeletier, 1836 | KJ848906 | KJ849052 | UFMG IHY 38314 | Dores do Rio Preto | Espírito Santo | Brazil |
| *Bombus brasiliensis* Lepeletier, 1836 | KJ848907 | KJ849053 | UFMG IHY 38317 | Dores do Rio Preto | Espírito Santo | Brazil |
| *Bombus brasiliensis* Lepeletier, 1836 | KJ848908 | KJ849054 | UFMG IHY 38318 | Dores do Rio Preto | Espírito Santo | Brazil |
| *Bombus brasiliensis* Lepeletier, 1836 | KJ848909 | KJ849055 | UFMG IHY 38320 | Dores do Rio Preto | Espírito Santo | Brazil |
| *Bombus pauloensis* Friese, 1913 | KJ848910 | KJ849056 | UFMG IHY 50317 | Patrocínio | Minas Gerais | Brazil |
| *Bombus brasiliensis* Lepeletier, 1836 | KJ848911 | KJ849057 | UFMG IHY 39095 | Baependi | Minas Gerais | Brazil |
| *Bombus brasiliensis* Lepeletier, 1836 | KJ848912 | KJ849058 | UFMG IHY 39100 | Baependi | Minas Gerais | Brazil |
| *Bombus brasiliensis* Lepeletier, 1836 | KJ848913 | KJ849059 | UFMG IHY 39104 | Baependi | Minas Gerais | Brazil |
| *Bombus brasiliensis* Lepeletier, 1836 | KJ848914 | KJ849060 | UFMG IHY 39105 | Baependi | Minas Gerais | Brazil |
| *Bombus brasiliensis* Lepeletier, 1836 | KJ848915 | KJ849061 | UFMG IHY 39106 | Baependi | Minas Gerais | Brazil |
| *Bombus brasiliensis* Lepeletier, 1836 | KJ848916 | KJ849062 | UFMG IHY 39107 | Baependi | Minas Gerais | Brazil |
| *Bombus brasiliensis* Lepeletier, 1836 | KJ848917 | KJ849063 | UFMG IHY 39025 | Aiuruoca | Minas Gerais | Brazil |
| *Bombus brasiliensis* Lepeletier, 1836 | KJ848918 | KJ849064 | UFMG IHY 39040 | Aiuruoca | Minas Gerais | Brazil |
| *Bombus brasiliensis* Lepeletier, 1836 | KJ848919 | KJ849065 | UFMG IHY 39054 | Aiuruoca | Minas Gerais | Brazil |
| *Bombus brasiliensis* Lepeletier, 1836 | KJ848920 | KJ849066 | UFMG IHY 39057 | Aiuruoca | Minas Gerais | Brazil |
| *Bombus pauloensis* Friese, 1913 | KJ848921 | KJ849067 | UFMG IHY 50318 | Patrocínio | Minas Gerais | Brazil |
| *Bombus brasiliensis* Lepeletier, 1836 | KJ848922 | KJ849068 | UFMG IHY 39058 | Aiuruoca | Minas Gerais | Brazil |
| *Bombus brasiliensis* Lepeletier, 1836 | KJ848923 | KJ849069 | UFMG IHY 39061 | Baependi | Minas Gerais | Brazil |
| *Bombus brasiliensis* Lepeletier, 1836 | KJ848924 | KJ849070 | UFMG IHY 39062 | Baependi | Minas Gerais | Brazil |
| *Bombus brasiliensis* Lepeletier, 1836 | KJ848925 | KJ849071 | UFMG IHY 39072 | Baependi | Minas Gerais | Brazil |
| *Bombus brasiliensis* Lepeletier, 1836 | KJ848926 | KJ849072 | UFMG IHY 39776 | Gonçalves | Minas Gerais | Brazil |
| *Bombus brasiliensis* Lepeletier, 1836 | KJ848927 | KJ849073 | UFMG IHY 39799 | Gonçalves | Minas Gerais | Brazil |
| *Bombus brasiliensis* Lepeletier, 1836 | KJ848928 | KJ849074 | UFMG IHY 39800 | Gonçalves | Minas Gerais | Brazil |
| *Bombus brasiliensis* Lepeletier, 1836 | KJ848929 | KJ849075 | UFMG IHY 39822 | Gonçalves | Minas Gerais | Brazil |
| *Bombus brasiliensis* Lepeletier, 1836 | KJ848930 | KJ849076 | UFMG IHY 39840 | Gonçalves | Minas Gerais | Brazil |
| *Bombus brasiliensis* Lepeletier, 1836 | KJ848931 | KJ849077 | UFMG IHY 41839 | Cororó | San Pedro | Paraguay |
| *Bombus morio* (Swederus, 1787) | KJ848932 |  | UFMG IHY 1075 | Belo Horizonte | Minas Gerais | Brazil |
| *Bombus brasiliensis* Lepeletier, 1836 | KJ848933 | KJ849078 | UFMG IHY | Ouro Preto | Minas Gerais | Brazil |
| *Bombus brasiliensis* Lepeletier, 1836 | KJ848934 | KJ849079 | UFMG IHY | Guaratuba | Paraná | Brazil |
| *Bombus brasiliensis* Lepeletier, 1836 | KJ848935 | KJ849080 | UFMG IHY | Guaratuba | Paraná | Brazil |
| *Bombus brasiliensis* Lepeletier, 1836 | KJ848936 | KJ849081 | UFMG IHY | Guaratuba | Paraná | Brazil |
| *Bombus brasiliensis* Lepeletier, 1836 | KJ848937 | KJ849082 | UFMG IHY | Guaratuba | Paraná | Brazil |
| *Bombus brasiliensis* Lepeletier, 1836 | KJ848938 | KJ849083 | UFMG IHY | Guaratuba | Paraná | Brazil |
| *Bombus morio* (Swederus, 1787) | KJ848939 |  | UFMG IHY 12338 | Nova Lima | Minas Gerais | Brazil |
| *Bombus brasiliensis* Lepeletier, 1836 | KJ848940 | KJ849084 | UFMG IHY | Guaratuba | Paraná | Brazil |
| *Bombus brasiliensis* Lepeletier, 1836 | KJ848941 | KJ849085 | UFMG IHY | Patrocínio | Minas Gerais | Brazil |
| *Bombus brasiliensis* Lepeletier, 1836 | KJ848942 | KJ849086 | UFMG IHY | Uberlândia | Minas Gerais | Brazil |
| *Bombus brasiliensis* Lepeletier, 1836 | KJ848943 | KJ849087 | UFMG IHY 39379 | Ouro Preto | Minas Gerais | Brazil |
| *Bombus bahiensis* sp. n. | KJ848944 | KJ849088 | UFMG IHY 52893 | Ilhéus | Bahia | Brazil |
| *Bombus bahiensis* sp. n. | KJ848945 | KJ849089 | UFMG IHY 52894 | Ilhéus | Bahia | Brazil |
| *Bombus transversalis* (Olivier, 1789) | KJ848946 |  | UFMG IHY | Guajará Mirim | Rondônia | Brazil |
| *Bombus transversalis* (Olivier, 1789) | KJ848947 | KJ849090 | UFMG IHY | Guajará Mirim | Rondônia | Brazil |
| *Bombus transversalis* (Olivier, 1789) | KJ848948 |  | UFMG IHY 23797 | Abel Figueiredo | Pará | Brazil |
| *Bombus transversalis* (Olivier, 1789) | KJ848949 |  | UFMG IHY 23798 | Abel Figueiredo | Pará | Brazil |
| *Bombus transversalis* (Olivier, 1789) | KJ848950 | KJ849091 | UFMG IHY 39349 | Alvorada do Oeste | Rondônia | Brazil |
| *Bombus brevivillus* Franklin, 1913 | KJ848951 |  | UFMG IHY 1207320 | Ubajara | Ceará | Brazil |
| *Bombus brevivillus* Franklin, 1913 | KJ848952 |  | UFMG IHY 1207321 | Ubajara | Ceará | Brazil |
| *Bombus brevivillus* Franklin, 1913 | KJ848953 |  | UFMG IHY 1207210 | Ubajara | Ceará | Brazil |
