# Supplementary material for: Hitting an Unintended Target: Phylogeography of Bombus brasiliensis Lepeletier, 1836 and the First New Brazilian Bumblebee Species in a Century (Hymenoptera: Apidae)
Source: PLoS One. 2015 May 20;10(5):e0125847. doi: 10.1371/journal.pone.0125847 (PMC4438978; doi:10.1371/journal.pone.0125847)
Supplement: S2 Table — (DOCX) [file pone.0125847.s003.docx]

**Table S2.**  **Protocol of the purification of Polyethylene Glycol 20% (PEG 20%) for elimination of bands <300-400 bp.**

| Step | Method Description |
| --- | --- |
| 01) | Transfer the PCR product to the tube 500μL; |
| 02) | Add the same volume of PEG solution (NaCl 2.5 M + PEG 20%) and vortex. |
| 03) | Let the PCR + PEG20% incubate at 37 °C for 15 minutes; |
| 04) | Centrifuge for 15 minutes at high speed (13,000 rpm); |
| 05) | Remove the supernatant and discard it; |
| 06) | Add 125 µl of cold 80% ethanol; |
| 07) | Centrifuge for 5 minutes at high speed (13,000 rpm); |
| 08) | Remove the supernatant and discard it; |
| 09) | Repeat step 06; |
| 10) | Leave to dry in an oven at 37 °C. There should be no trace of ethanol when done; |
| 11) | Dissolve the PCR product in milliQ water. The volume should be proportional to the initial concentration of DNA. |
