# Supplementary material for: Hitting an Unintended Target: Phylogeography of Bombus brasiliensis Lepeletier, 1836 and the First New Brazilian Bumblebee Species in a Century (Hymenoptera: Apidae)
Source: PLoS One. 2015 May 20;10(5):e0125847. doi: 10.1371/journal.pone.0125847 (PMC4438978; doi:10.1371/journal.pone.0125847)
Supplement: S3 Table — The model used was the Kimura 2-parameter. Bees currently considered as B. brevivillus belong in two distinct species, identified below as B. brevivillus (1) and B. brevivillus (2). AD = Average interspecific distance ± standard deviation; MiD = minimum interespecific distance ± standard deviation. (DOCX) [file pone.0125847.s004.docx]

**Table S3. Genetic distances for COI sequences (%) between bumblebee-species. The model used was the Kimura 2-parameter.** Bees currently considered as *B. brevivillus* belong in two distinct species, identified below as *B. brevivillus* (1) and *B. brevivillus* (2). AD = Average interspecific distance ± standard deviation; MiD = minimum interespecific distance ± standard deviation.

| Taxon 1 | Taxon 2 | AD | MiD |
| --- | --- | --- | --- |
| *B. bahiensis* sp. n. | *B. bellicosus* | 4.39±1.00 | 4.39±1.00 |
| *B. bahiensis* sp. n. | *B. brevivillus* (1) | 4.12±1.00 | 4.12±0.98 |
| *B. bahiensis* sp. n. | *B. brevivillus* (2) | 6.98±1.32 | 6.79±1,32 |
| *B. bahiensis* sp. n. | *B. deuteronymus* | 8.14±1.41 | 8.14±1.41 |
| *B. bahiensis* sp. n. | *B. diligens* | 4.91±1.08 | 4.91±1.12 |
| *B. bahiensis* sp. n. | *B. excellens* | 15.41±2.10 | 15.41±2.13 |
| *B. bahiensis* sp. n. | *B. fervidus* | 5.18±1.09 | 5.18±1.13 |
| *B. bahiensis* sp. n. | *B. humilis* | 9.26±1.49 | 9.26±1.51 |
| *B. bahiensis* sp. n. | *B. medius* | 5.71±1.18 | 5.71±1.18 |
| *B. bahiensis* sp. n. | *B. mesomelas* | 8.97±1.50 | 8.97±1.49 |
| *B. bahiensis* sp. n. | *B. muscorum* | 10.10±1.61 | 10.10±1.65 |
| *B. bahiensis* sp. n. | *B. opifex* | 5.16±1.08 | 5.16±1.09 |
| *B. bahiensis* sp. n. | *B. pascuorum* | 9.28±1.53 | 8.97±1.53 |
| *B. bahiensis* sp. n. | *B. pensylvanicus* | 6.23±1.23 | 6.23±1.25 |
| *B. bahiensis* sp. n. | *B. ruderarius* | 8.97±1.52 | 8.97±1.47 |
| *B. bahiensis* sp. n. | *B. schrencki* | 9.54±1.55 | 9.54±1.56 |
| *B. bahiensis* sp. n. | *B. sylvarum* | 9.54±1.58 | 9.54±1.57 |
| *B. bahiensis* sp. n. | *B. transversalis* | 4.43±1.02 | 4.37±1.02 |
| *B. bellicosus* | *B. brevivillus* (2) | 6.97±1.26 | 7.06±1.30 |
| *B. bellicosus* | *B. deuteronymus* | 7.04±1.29 | 7.04±1.30 |
| *B. bellicosus* | *B. diligens* | 3.58±0.95 | 3.58±0.96 |
| *B. bellicosus* | *B. excellens* | 13.80±1.98 | 13.60±2.00 |
| *B. bellicosus* | *B. fervidus* | 4.36±1.01 | 4.36±1.04 |
| *B. bellicosus* | *B. humilis* | 8.14±1.41 | 8.14±1.44 |
| *B. bellicosus* | *B. medius* | 4.90±1.09 | 4.90±1.12 |
| *B. bellicosus* | *B. mesomelas* | 7.68±1.41 | 7.59±1.40 |
| *B. bellicosus* | *B. muscorum* | 8.42±1.43 | 8.42±1.49 |
| *B. bellicosus* | *B. opifex* | 3.57±0.91 | 3.57±0.93 |
| *B. bellicosus* | *B. pascuorum* | 8.17±1.41 | 7.86±1.43 |
| *B. bellicosus* | *B. pensylvanicus* | 4.89±1.10 | 4.89±1.11 |
| *B. bellicosus* | *B. ruderarius* | 7.86±1.38 | 7.86±1.38 |
| *B. bellicosus* | *B. schrencki* | 8.70±1.47 | 8.70±1.51 |
| *B. bellicosus* | *B. sylvarum* | 8.14±1.41 | 8.14±1.41 |
| *B. brasiliensis* | *B. bahiensis* sp. n. | 2.89±0.80 | 2.55±0.78 |
| *B. brasiliensis* | *B. bellicosus* | 5.08±1.12 | 4.65±1.10 |
| *B. brasiliensis* | *B. brevivillus* (1) | 3.74±0.96 | 3.32±0.93 |
| *B. brasiliensis* | *B. brevivillus* (2) | 7.02±1.31 | 6.51±1.27 |
| *B. brasiliensis* | *B. deuteronymus* | 8.01±1.38 | 7.59±1.36 |
| *B. brasiliensis* | *B. diligens* | 5.06±1.12 | 4.63±1.12 |
| *B. brasiliensis* | *B. excellens* | 15.90±2.16 | 15.41±2.15 |
| *B. brasiliensis* | *B. fervidus* | 5.31±1.09 | 4.90±1.10 |
| *B. brasiliensis* | *B. humilis* | 9.69±1.52 | 9.26±1.50 |
| *B. brasiliensis* | *B. medius* | 5.83±1.21 | 5.43±1.28 |
| *B. brasiliensis* | *B. mesomelas* | 8.56±1.44 | 8.14±1.40 |
| *B. brasiliensis* | *B. morio* | 11.20±1.66 | 10.68±1.65 |
| *B. brasiliensis* | *B. muscorum* | 9.97±1.59 | 9.54±1.59 |
| *B. brasiliensis* | *B. opifex* | 5.58±1.16 | 5.15±1.14 |
| *B. brasiliensis* | *B. pascuorum* | 9.43±1.55 | 8.70±1.52 |
| *B. brasiliensis* | *B. pensylvanicus* | 6.39±1.25 | 5.96±1.26 |
| *B. brasiliensis* | *B. ruderarius* | 8.56±1.47 | 8.14±1.42 |
| *B. brasiliensis* | *B. schrencki* | 9.97±1.56 | 9.54±1.56 |
| *B. brasiliensis* | *B. sylvarum* | 9.14±1.54 | 8.69±1.51 |
| *B. brasiliensis* | *B. transversalis* | 4.58±1.03 | 4.10±1.00 |
| *B. brevivillus* (1) | *B. bellicosus* | 3.58±0.92 | 6.78±1.29 |
| *B. brevivillus* (1) | *B. brevivillus* (2) | 3.92±0.93 | 3.83±0.92 |
| *B. brevivillus* (1) | *B. deuteronymus* | 7.59±1.34 | 7.59±1.34 |
| *B. brevivillus* (1) | *B. diligens* | 4.36±1.03 | 4.36±1.09 |
| *B. brevivillus* (1) | *B. excellens* | 14.20±2.05 | 14.20±2.10 |
| *B. brevivillus* (1) | *B. fervidus* | 4.63±1.05 | 4.63±1.08 |
| *B. brevivillus* (1) | *B. humilis* | 8.70±1.46 | 8.70±1.48 |
| *B. brevivillus* (1) | *B. medius* | 5.16±1.13 | 5.16±1.14 |
| *B. brevivillus* (1) | *B. mesomelas* | 8.70±1.49 | 8.70±1.49 |
| *B. brevivillus* (1) | *B. muscorum* | 8.97±1.50 | 8.97±1.54 |
| *B. brevivillus* (1) | *B. opifex* | 4.89±1.08 | 4.89±1.11 |
| *B. brevivillus* (1) | *B. pascuorum* | 9.00±1.48 | 8.70±1.50 |
| *B. brevivillus* (1) | *B. pensylvanicus* | 5.15±1.16 | 5.15±1.21 |
| *B. brevivillus* (1) | *B. ruderarius* | 8.69±1.49 | 8.69±1.49 |
| *B. brevivillus* (1) | *B. schrencki* | 9.54±1.53 | 9.54±1.56 |
| *B. brevivillus* (1) | *B. sylvarum* | 9.26±1.53 | 9.26±1.52 |
| *B. brevivillus* (1) | *B. transversalis* | 3.36±0.90 | 3.31±0.94 |
| *B. brevivillus* (2) | *B. deuteronymus* | 9.64±1.54 | 9.54±1.50 |
| *B. brevivillus* (2) | *B. diligens* | 7.69±1.36 | 7.60±1.40 |
| *B. brevivillus* (2) | *B. excellens* | 17.06±2.20 | 16.96±2.23 |
| *B. brevivillus* (2) | *B. fervidus* | 6.61±1.25 | 6.52±1.26 |
| *B. brevivillus* (2) | *B. humilis* | 10.58±1.62 | 10.39±1.58 |
| *B. brevivillus* (2) | *B. medius* | 8.52±1.46 | 8.43±1.46 |
| *B. brevivillus* (2) | *B. mesomelas* | 9.17±1.54 | 8.98±1.48 |
| *B. brevivillus* (2) | *B. muscorum* | 10.30±1.59 | 10.11±1.58 |
| *B. brevivillus* (2) | *B. opifex* | 8.52±1.39 | 8.43±1.40 |
| *B. brevivillus* (2) | *B. pascuorum* | 10.32±1.61 | 10.10±1.61 |
| *B. brevivillus* (2) | *B. pensylvanicus* | 8.24±1.44 | 8.15±1.49 |
| *B. brevivillus* (2) | *B. ruderarius* | 10.58±1.66 | 10.39±1.60 |
| *B. brevivillus* (2) | *B. schrencki* | 10.87±1.66 | 10.68±1.65 |
| *B. brevivillus* (2) | *B. sylvarum* | 12.32±1.77 | 12.12±1.75 |
| *B. deuteronymus* | *B. diligens* | 6.78±1.27 | 6.78±1.29 |
| *B. deuteronymus* | *B. excellens* | 13.61±1.93 | 13.61±1.96 |
| *B. deuteronymus* | *B. humilis* | 2.53±0.76 | 2.53±0.74 |
| *B. deuteronymus* | *B. medius* | 7.31±1.34 | 7.31±1.34 |
| *B. deuteronymus* | *B. mesomelas* | 7.06±1.34 | 7.06±1.37 |
| *B. deuteronymus* | *B. opifex* | 5.70±1.14 | 5.70±1.13 |
| *B. deuteronymus* | *B. pensylvanicus* | 8.15±1.41 | 8.15±1.44 |
| *B. deuteronymus* | *B. ruderarius* | 3.05±0.87 | 3.05±0.87 |
| *B. deuteronymus* | *B. sylvarum* | 4.89±1.10 | 4.89±1.11 |
| *B. diligens* | *B. excellens* | 13.31±1.98 | 13.31±2.00 |
| *B. diligens* | *B. medius* | 4.89±1.10 | 4.89±1.08 |
| *B. diligens* | *B. opifex* | 4.09±0.99 | 4.09±1.00 |
| *B. diligens* | *B. pensylvanicus* | 4.35±1.04 | 4.35±1.04 |
| *B. excellens* | *B. medius* | 14.80±2.05 | 14.80±2.08 |
| *B. excellens* | *B. opifex* | 13.31±1.91 | 13.31±1.97 |
| *B. excellens* | *B. pensylvanicus* | 12.42±1.87 | 12.42±1.89 |
| *B. fervidus* | *B. deuteronymus* | 7.04±1.30 | 7.04±1.29 |
| *B. fervidus* | *B. diligens* | 4.35±1.02 | 4.35±1.04 |
| *B. fervidus* | *B. excellens* | 15.42±2.11 | 15.42±2.14 |
| *B. fervidus* | *B. humilis* | 7.59±1.34 | 7.59±1.37 |
| *B. fervidus* | *B. medius* | 4.89±1.09 | 4.89±1.15 |
| *B. fervidus* | *B. mesomelas* | 6.50±1.27 | 6.50±1.27 |
| *B. fervidus* | *B. opifex* | 4.88±1.05 | 4.88±1.11 |
| *B. fervidus* | *B. pascuorum* | 6.79±1.30 | 6.79±1.30 |
| *B. fervidus* | *B. pensylvanicus* | 4.62±1.06 | 4.62±1.10 |
| *B. fervidus* | *B. ruderarius* | 7.04±1.32 | 7.04±1.28 |
| *B. fervidus* | *B. schrencki* | 7.31±1.31 | 7.31±1.35 |
| *B. fervidus* | *B. sylvarum* | 8.14±1.42 | 8.14±1.42 |
| *B. humilis* | *B. diligens* | 8.16±1.42 | 8.16±1.46 |
| *B. humilis* | *B. excellens* | 12.72±1.82 | 12.72±1.85 |
| *B. humilis* | *B. medius* | 8.42±1.46 | 8.42±1.53 |
| *B. humilis* | *B. opifex* | 6.24±1.22 | 6.24±1.25 |
| *B. humilis* | *B. pensylvanicus* | 9.28±1.53 | 9.28±1.57 |
| *B. medius* | *B. pensylvanicus* | 5.68±1.15 | 5.68±1.17 |
| *B. mesomelas* | *B. diligens* | 7.60±1.40 | 7.60±1.37 |
| *B. mesomelas* | *B. excellens* | 14.84±2.05 | 14.84±2.08 |
| *B. mesomelas* | *B. humilis* | 6.24±1.23 | 6.24±1.26 |
| *B. mesomelas* | *B. medius* | 8.42±1.51 | 8.42±1.52 |
| *B. mesomelas* | *B. opifex* | 7.33±1.36 | 7.33±1.41 |
| *B. mesomelas* | *B. pensylvanicus* | 8.15±1.44 | 8.15±1.48 |
| *B. morio* | *B. bahiensis* sp. n. | 11.04±1.66 | 10.96±1.70 |
| *B. morio* | *B. bellicosus* | 9.61±1.58 | 9.54±1.60 |
| *B. morio* | *B. brevivillus* (1) | 10.75±1.65 | 10.68±1.68 |
| *B. morio* | *B. brevivillus* (2) | 13.14±1.82 | 12.42±1.78 |
| *B. morio* | *B. deuteronymus* | 10.46±1.64 | 10.13±1.65 |
| *B. morio* | *B. diligens* | 9.33±1.53 | 9.26±1.57 |
| *B. morio* | *B. excellens* | 14.58±1.99 | 13.90±1.92 |
| *B. morio* | *B. fervidus* | 9.56±1.52 | 9.26±1.54 |
| *B. morio* | *B. humilis* | 10.17±1.59 | 9.84±1.62 |
| *B. morio* | *B. medius* | 10.13±1.59 | 9.54±1.59 |
| *B. morio* | *B. mesomelas* | 8.72±1.46 | 8.42±1.46 |
| *B. morio* | *B. muscorum* | 10.45±1.65 | 10.12±1.67 |
| *B. morio* | *B. opifex* | 8.21±1.43 | 8.14±1.50 |
| *B. morio* | *B. pascuorum* | 9.91±1.55 | 9.27±1.57 |
| *B. morio* | *B. pensylvanicus* | 7.43±1.34 | 7.31±1.36 |
| *B. morio* | *B. ruderarius* | 11.32±1.70 | 10.98±1.71 |
| *B. morio* | *B. schrencki* | 10.50±1.60 | 10.12±1.62 |
| *B. morio* | *B. sylvarum* | 9.59±1.54 | 9.26±1.53 |
| *B. morio* | *B. transversalis* | 9.89±1.58 | 9.82±1.59 |
| *B. muscorum* | *B. deuteronymus* | 4.10±1.02 | 4.10±1.04 |
| *B. muscorum* | *B. diligens* | 7.33±1.33 | 7.33±1.38 |
| *B. muscorum* | *B. excellens* | 13.61±1.98 | 13.61±2.01 |
| *B. muscorum* | *B. fervidus* | 7.87±1.38 | 7.87±1.42 |
| *B. muscorum* | *B. humilis* | 3.31±0.87 | 3.31±0.90 |
| *B. muscorum* | *B. medius* | 8.42±1.44 | 8.42±1.51 |
| *B. muscorum* | *B. mesomelas* | 7.06±1.31 | 7.06±1.35 |
| *B. muscorum* | *B. opifex* | 7.61±1.38 | 7.61±1.42 |
| *B. muscorum* | *B. pascuorum* | 3.08±0.82 | 2.78±0.81 |
| *B. muscorum* | *B. pensylvanicus* | 8.99±1.49 | 8.99±1.52 |
| *B. muscorum* | *B. ruderarius* | 4.36±1.04 | 4.36±1.08 |
| *B. muscorum* | *B. schrencki* | 4.10±1.01 | 4.10±1.02 |
| *B. muscorum* | *B. sylvarum* | 4.89±1.06 | 4.89±1.06 |
| *B. opifex* | *B. medius* | 5.42±1.16 | 5.42±1.19 |
| *B. opifex* | *B. pensylvanicus* | 5.42±1.16 | 5.42±1.20 |
| *B. pascuorum* | *B. deuteronymus* | 3.33±0.90 | 3.05±0.88 |
| *B. pascuorum* | *B. diligens* | 7.35±1.35 | 7.05±1.36 |
| *B. pascuorum* | *B. excellens* | 12.51±1.83 | 12.42±1.85 |
| *B. pascuorum* | *B. humilis* | 1.84±0.64 | 1.76±0.63 |
| *B. pascuorum* | *B. medius* | 8.17±1.43 | 7.87±1.46 |
| *B. pascuorum* | *B. mesomelas* | 6.54±1.26 | 6.24±1.29 |
| *B. pascuorum* | *B. opifex* | 6.00±1.18 | 5.70±1.16 |
| *B. pascuorum* | *B. pensylvanicus* | 7.90±1.42 | 7.60±1.42 |
| *B. pascuorum* | *B. ruderarius* | 2.35±0.76 | 2.27±0.79 |
| *B. pascuorum* | *B. schrencki* | 2.04±0.69 | 1.76±0.64 |
| *B. pascuorum* | *B. sylvarum* | 4.44±1.05 | 4.36±1.06 |
| *B. pauloensis* | *B. bahiensis* sp. n. | 4.60±1.03 | 4.37±1.02 |
| *B. pauloensis* | *B. bellicosus* | 4.87±1.10 | 4.63±1.07 |
| *B. pauloensis* | *B. brasiliensis* | 3.48±0.85 | 2.79±0.83 |
| *B. pauloensis* | *B. brevivillus* (1) | 3.96±0.97 | 3.83±0.99 |
| *B. pauloensis* | *B. brevivillus* (2) | 7.55±1.34 | 6.77±1.30 |
| *B. pauloensis* | *B. deuteronymus* | 8.10±1.39 | 7.87±1.40 |
| *B. pauloensis* | *B. diligens* | 4.88±1.08 | 4.62±1.08 |
| *B. pauloensis* | *B. excellens* | 14.15±2.05 | 13.90±2.06 |
| *B. pauloensis* | *B. fervidus* | 5.11±1.07 | 4.88±1.09 |
| *B. pauloensis* | *B. humilis* | 8.66±1.44 | 8.43±1.46 |
| *B. pauloensis* | *B. medius* | 5.64±1.16 | 5.15±1.12 |
| *B. pauloensis* | *B. mesomelas* | 7.54±1.32 | 7.32±1.32 |
| *B. pauloensis* | *B. morio* | 8.44±1.40 | 8.14±1.39 |
| *B. pauloensis* | *B. muscorum* | 9.48±1.52 | 9.27±1.57 |
| *B. pauloensis* | *B. opifex* | 5.42±1.14 | 5.15±1.16 |
| *B. pauloensis* | *B. pascuorum* | 8.97±1.49 | 8.43±1.50 |
| *B. pauloensis* | *B. pensylvanicus* | 5.64±1.14 | 5.41±1.14 |
| *B. pauloensis* | *B. ruderarius* | 8.70±1.47 | 8.42±1.47 |
| *B. pauloensis* | *B. schrencki* | 9.50±1.51 | 9.27±1.53 |
| *B. pauloensis* | *B. sylvarum* | 8.68±1.47 | 8.42±1.46 |
| *B. pauloensis* | *B. transversalis* | 4.41±1.03 | 4.09±1.00 |
| *B. ruderarius* | *B. diligens* | 7.60±1.39 | 7.60±1.36 |
| *B. ruderarius* | *B. excellens* | 13.91±1.98 | 13.91±1.99 |
| *B. ruderarius* | *B. humilis* | 2.53±0.80 | 2.53±0.79 |
| *B. ruderarius* | *B. medius* | 7.86±1.44 | 7.86±1.44 |
| *B. ruderarius* | *B. mesomelas* | 5.69±1.15 | 5.69±1.18 |
| *B. ruderarius* | *B. opifex* | 6.24±1.21 | 6.24±1.19 |
| *B. ruderarius* | *B. pensylvanicus* | 8.71±1.51 | 8.71±1.50 |
| *B. schrencki* | *B. deuteronymus* | 3.31±0.90 | 3.31±0.87 |
| *B. schrencki* | *B. diligens* | 8.43±1.46 | 8.43±1.50 |
| *B. schrencki* | *B. excellens* | 13.31±1.90 | 13.31±1.91 |
| *B. schrencki* | *B. humilis* | 2.27±0.73 | 2.27±0.74 |
| *B. schrencki* | *B. medius* | 8.70±1.49 | 8.70±1.52 |
| *B. schrencki* | *B. mesomelas* | 6.51±1.24 | 6.51±1.29 |
| *B. schrencki* | *B. opifex* | 6.51±1.24 | 6.51±1.26 |
| *B. schrencki* | *B. pensylvanicus* | 8.43±1.44 | 8.43±1.46 |
| *B. schrencki* | *B. ruderarius* | 3.31±0.88 | 3.31±0.89 |
| *B. schrencki* | *B. sylvarum* | 4.88±1.07 | 4.88±1.08 |
| *B. sylvarum* | *B. diligens* | 7.88±1.41 | 7.88±1.41 |
| *B. sylvarum* | *B. excellens* | 14.81±2.02 | 14.81±2.03 |
| *B. sylvarum* | *B. humilis* | 4.62±1.07 | 4.62±1.09 |
| *B. sylvarum* | *B. medius* | 8.42±1.46 | 8.42±1.50 |
| *B. sylvarum* | *B. mesomelas* | 7.88±1.42 | 7.88±1.44 |
| *B. sylvarum* | *B. opifex* | 6.78±1.29 | 6.78±1.28 |
| *B. sylvarum* | *B. pensylvanicus* | 8.71±1.50 | 8.71±1.48 |
| *B. sylvarum* | *B. ruderarius* | 4.09±1.02 | 4.09±1.03 |
| *B. transversalis* | *B. bellicosus* | 4.15±1.02 | 4.10±1.00 |
| *B. transversalis* | *B. brevivillus* (2) | 6.92±1.27 | 6.77±1.31 |
| *B. transversalis* | *B. deuteronymus* | 7.65±1.33 | 7.60±1.34 |
| *B. transversalis* | *B. diligens* | 2.58±0.76 | 2.53±0.77 |
| *B. transversalis* | *B. excellens* | 13.96±2.00 | 13.90±2.03 |
| *B. transversalis* | *B. fervidus* | 4.41±1.03 | 4.36±1.06 |
| *B. transversalis* | *B. humilis* | 9.05±1.47 | 8.99±1.51 |
| *B. transversalis* | *B. medius* | 4.94±1.06 | 4.88±1.06 |
| *B. transversalis* | *B. mesomelas* | 8.48±1.47 | 8.43±1.44 |
| *B. transversalis* | *B. muscorum* | 8.76±1.47 | 8.70±1.49 |
| *B. transversalis* | *B. opifex* | 4.62±1.07 | 4.62±1.06 |
| *B. transversalis* | *B. pascuorum* | 8.23±1.41 | 7.87±1.40 |
| *B. transversalis* | *B. pensylvanicus* | 4.14±1.02 | 4.09±1.05 |
| *B. transversalis* | *B. ruderarius* | 8.48±1.44 | 8.42±1.42 |
| *B. transversalis* | *B. schrencki* | 9.32±1.52 | 9.27±1.54 |
| *B. transversalis* | *B. sylvarum* | 9.04±1.53 | 8.98±1.52 |
